# Setting Policy Priorities for Front-of-Pack Health Claims and Symbols in the European Union: Expert Consensus Built by Using a Delphi Method

**DOI:** 10.3390/nu11020403

**Published:** 2019-02-14

**Authors:** Yung Hung, Sophie Hieke, Klaus G Grunert, Wim Verbeke

**Affiliations:** 1 Department of Agricultural Economics, Ghent University, Coupure links 653, 9000 Ghent, Belgium; wim.verbeke@ugent.be; 2 European Food Information Council (EUFIC), Rue des Deux Eglises 14 (3rd floor), 1000 Brussels, Belgium; sophie.hieke@eufic.org; 3 MAPP Centre, Aarhus University, Bartholins Allé 10, 8000 Aarhus C, Denmark; klg@mgmt.au.dk

**Keywords:** nutrition label, health claims, health symbols, consumer behavior, public health policy, communication, delphi method

## Abstract

Despite the fact that front-of-pack nutrition labels such as health claims and symbols have received growing attention in consumer behavior research, comprehensive conclusions could not yet be drawn to develop concrete policy actions, owing to the complexity of the subject and a constantly changing market environment. In this study, evidence-based policy recommendations and communication guidelines have been derived from the findings of the EU FP7 project CLYMBOL (“Role of health-related CLaims and sYMBOLs in consumer behavior”, Grant Agreement 311963), and have been evaluated and prioritized by European stakeholders using a three-round Delphi method. A moderate level of consensus was achieved and results suggest that policy priority should focus on ways to improve consumer motivation and interest in healthy eating. Consumers’ interest in healthy eating could be increased by adopting appropriate communication strategies such as using innovative ways to communicate the importance of healthy eating, which may aim to change the possible negative association between healthiness and tastiness. The highest-rated finding was related to consumers’ favorable attitude towards health claims with shorter and less complex messages and health symbols with a visible endorsement. Meanwhile, there was a clear consensus that health claims need to be scientifically substantiated and credible but phrased without using overly complex scientific wordings, in order to be meaningful for consumers. Furthermore, stakeholders from academia and industry believe that consumer awareness about existing health claims should be increased. The identified policy recommendations and communication guidelines stem from recent empirical evidence and provide useful insights that guide future policy development aligning consumer protection issues as well as public health and food marketing communication interests.

## 1. Introduction

Effective public policies are essential to improve food environments for consumers. European Union (EU) legislation (EC 1924/2006) has harmonized the use of health claims and symbols with more clearly established rules and built-in safeguards against misleading consumers (European Commission, 2006). With public health goals in mind, health claims and symbols are expected to support consumers in making more informed and healthier food choices, as well as foster industry competitiveness. After a decade of being in effect, it opens up the discussion of whether this legislation has achieved its intended effects. The majority of nutrition and health claims and all health symbols are on the front-of-pack (FOP) [1], aiming to provide consumers with relevant health information in a format that is appealing and easy to access and process [2]. Although the role of health claims and symbols in consumer behavior has received increasing attention in research [3,4], comprehensive conclusions for meaningful policy recommendations could not yet be drawn, due to complexity of the subject and a constantly changing market environment [5,6]. The EU-funded FP7 project CLYMBOL has envisaged providing wide-ranging assessments of the role of health claims and symbols on food and drink products in consumers’ food choices, and to derive evidence-based implications for future policy development and communication. The project has covered four main work areas. First, the current status of health claims and symbols has been reviewed, providing an overview of the history of health claim and symbol use and its prevalence on foods across the EU Member States [1,4,7,8]. Second, consumers’ needs and wants in relation to foods with health claims and symbols have been studied, explaining how consumers perceive and use health claims and symbols based on their perceptions, processing motivation and ability [9,10,11,12,13,14]. Third, a methodological toolbox has been developed, offering a set of tested methods and strategies for answering future research questions [11,15,16,17]. Fourth, effects of health claims and symbols on consumer understanding, purchase and consumption have been investigated [18,19,20,21]. These assessments employed a wide range of empirical and methodological studies, ranging from interviews, online surveys, laboratory experiments, and in-store tests to econometric modelling of household panel data and product sampling, in order to yield comprehensive evidence while accounting the underlying complexities such as cultural differences and individual consumer characteristics. The four work areas have been described in detail in [22].

The present study prioritizes qualitative insights using quantitative data. Key findings from earlier studies within the four CLYMBOL work areas formed the basis for formulating evidence-based policy recommendations and communication guidelines. Since these recommendations and guidelines were formed for EU policy directed towards the regulation and provision of health claims and symbols, active involvement of stakeholders from various sectors was required to warrant meaningful insights. Specific questions were to what extent recommendations and communication guidelines are relevant to the stakeholder’s organization and how feasible they rate their implementation in practice. This has been studied using the Delphi method. Formal expert elicitation through the Delphi method is a structured communication process that allows a heterogeneous group of experts to share views, build consensus, and deal with a complex problem [23,24]. One of the advantages of the Delphi method is that the aggregated response from a group of experts is expected to be less prone to “mistakes” [25]. This method has been applied for policy planning since the 1970s [26], and it remains popular in public food and health policy development [27,28].

This study is, to our best knowledge, the first attempt to formulate an exhaustive list of policy recommendations and communication guidelines for future development of FOP nutrition labels such as health claims and symbols, based on key findings from more than 20 empirical studies undertaken across EU Member States. These qualitative recommendations and guidelines were then evaluated by stakeholders through three Delphi rounds and prioritized using quantitative data. Our study aims to provide decision makers and researchers with a policy priority list and appropriate strategies that can guide future policy development aligning consumer protection issues, as well as public health and food marketing communication interests.

## 2. Materials and Methods

### 2.1. Procedure

**Figure 1:**
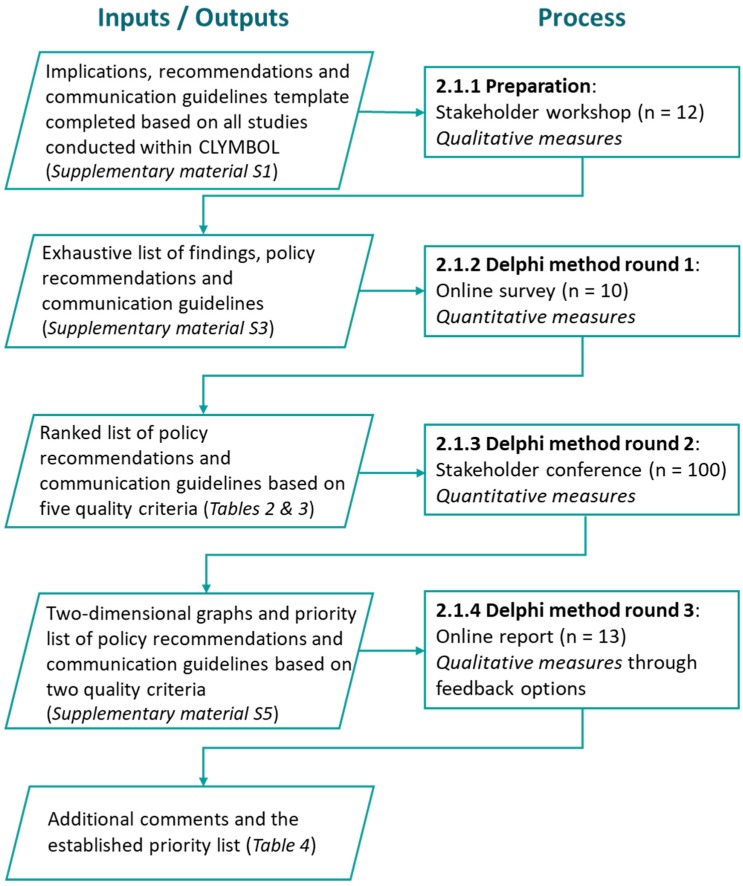
Flow chart of the Delphi study rounds and the corresponding inputs and outputs.

This Delphi study consisted of three rounds, in which stakeholders participated in person and/or online. After each round, an anonymous summary of results from the previous round was presented to the participants so they could compare these with their own views [29]. During this iterative process, the diversity of responses decreased and the group converged towards a consensus of views and opinions [30]. Prior to the study, ethics approval was granted by the Belgian Ethics Committee of Ghent University Hospital on 10/05/2016 (Reference No. B670201628360). Figure 1 provides a flow chart that illustrates the full procedure of the Delphi method and the inputs or outputs involved.

#### 2.1.1. Preparation

A template of Implications, Recommendations and Communication guidelines (further referred to as IRCs) was drafted (Supplementary Material S1) and completed alongside each CLYMBOL study by the directly involved researchers and checked by all project partners. The initial inputs were shared before and presented during a stakeholder workshop organized on 23 September 2015 in Copenhagen, Denmark. The participants were members of the CLYMBOL stakeholder advisory broad, which consisted of representatives from national food authorities, consumer and patient organizations and (food) industry associations (*n* = 12). They were closely involved throughout the entire project, but more specifically also in this study from the testing of initial ideas to participation in the actual Delphi study. In this stakeholder workshop, participants were divided into three groups according to their fields of activity, and each group had an even distribution of different types of stakeholder. They were invited to discuss and state opinions about the preliminary list of IRC. Based on their comments, the IRCs were refined prior to starting the first Delphi method round.

#### 2.1.2. Delphi Round 1

A list of findings, policy recommendations, and communication guidelines was compiled from the IRCs and structured based on the four work areas of the CLYMBOL project. This exhaustive list was first distributed to the stakeholders by e-mail, then the stakeholders were invited to evaluate all items through an online survey, keeping in mind the overall objective of prioritizing policy recommendations and communication guidelines, which was clearly stated in this survey as follows: “To support consumers in making informed and healthy food choices and foster industry competitiveness, taking into account individual and country differences within the EU, as well as to avoid misunderstanding and undesirable behavioral effects”.

The CLYMBOL findings were evaluated with a score from 1–10 on the basis of five criteria: relevance (1 = absolutely irrelevant; 10 = absolutely relevant); importance (1 = absolutely unimportant; 10 = absolutely important); novelty (1 = absolutely old finding(s); 10 = absolutely novel finding(s)); clarity (1 = absolutely unclear; 10 = absolutely clear); and consistency with your own belief (1 = absolutely contradictory to belief; 10 = absolutely consistent with belief). The findings dealing with the same variable(s) of interest were grouped. These CLYMBOL findings were included as part of the evaluation in order to inform stakeholders about the empirical evidence that had informed the respective recommendations and guidelines. Nevertheless, as it was not the focus of this study to evaluate findings from earlier studies, and neither was it the scope of the present paper, the evaluation outcomes of the CLYMBOL findings were not further evaluated in the next Delphi method rounds.

Policy recommendations and communication guidelines were also evaluated with a score from 1–10 on five criteria: feasibility (1 = absolutely unfeasible; 10 = absolutely feasible); effectiveness (defined as capability of producing desired results) (1 = absolutely ineffective; 10 = absolutely effective); efficiency (defined as the ratio between desired results and inputs of resources) (1 = absolutely inefficient; 10 = absolutely efficient); coherence with current policy in your organization (1 = absolutely contradictory to current policies; 10 = absolutely coherent with current policies); and unlikelihood of negative impacts (1 = absolutely likely to have negative impacts; 10 = absolutely unlikely to have negative impacts). Based on the scores given, a ranking could be allocated to each (group of) item(s), computed by averaging scores from the five evaluation criteria.

In total, there were 39 items of findings, 34 items of policy recommendations, and 22 items of communication guidelines derived from the earlier CLYMBOL studies, most of which have been published in (or are under consideration for publication in) scientific literature (www.clymbol.eu/outcomes/publications). In order to shorten this list and enhance the readability and consistency of interpretation, some items were grouped based on the corresponding findings. The policy recommendations or communication guidelines that were supported by multiple findings were only displayed once, so the stakeholders did not need to evaluate the same item multiple times. As a result, there were finally 22 (groups of) items of findings, 22 (groups of) items of policy recommendations and 17 (groups of) items of communication guidelines in the Delphi method round 1 evaluation, which was completed by 10 stakeholders.

#### 2.1.3. Delphi Round 2

The CLYMBOL stakeholder conference “Consumers and health claims” was organized on 15 June 2016 in Brussels, Belgium. The main project findings were presented and discussed during the event, and the results from the Delphi method round 1 were disseminated among the audience. The items of policy recommendations and communication guidelines with the highest mean scores based on the five criteria were presented item by item in descending order of scoring.

All conference attendees were invited to evaluate the items of policy recommendations and communication guidelines using a real-time voting system (Turning Technologies, LLC Youngstown, Ohio, USA) with a score from 1–7 based on two criteria: relevance to your organization (1 = absolutely irrelevant to your organization; 7 = absolutely relevant to your organization); and feasibility in practice (1 = absolutely unfeasible in practice; 7 = absolutely feasible in practice). These two criteria are hereafter referred to as “relevance” and “feasibility”. Communication guideline **items xiv** and **xv** were evaluated together as one item in the Delphi rounds, thus they were combined into one item namely **item xiv**. Due to a time constraint at the stakeholder conference, evaluation in the Delphi round two was based on less quality criteria (i.e., five vs. two) and point interval scales (i.e., ten vs. seven), compared to the Delphi round 1. Earlier studies suggested that 7-point interval scales performed significantly better in Delphi studies than other scales [31,32]. The live voting was completed by about 100 stakeholders from various sectors (Table 1). Presentation slides used for the real-time voting are available in Supplementary Material S2. 

The stakeholders who participated in the Delphi method round 2 (*n* = 100) were categorized into three groups for meaningful statistical comparison: industry (*n* = 38, i.e., food producer, retailer and food industry associations); academia (*n* = 25, i.e., academia and research institutes); and others (*n* = 37, i.e., government, NGO, legal adviser, consumer organization, health professional and others). The three groups mainly represent different interests or points of view, i.e., industry is expected to have a more commercial interest, academia to hold more science-based nuanced opinions, and the others may hold a more consumer protection-focused interest.

#### 2.1.4. Delphi Round 3

Results of the live voting from the Delphi method round 2 were presented to all stakeholders who participated in the previous round (the June 2016 stakeholder conference and online voting) through an online survey by means of a short report during July 2016. The Delphi round 3 was intended to confirm the voting outcomes obtained in the Delphi round 2 by informing and providing stakeholders with results and an opportunity to comment on each item and the corresponding relevance-feasibility graph. Stakeholders were invited through e-mail (with a link to the survey) to read the report and provide comments or feedback regarding the results (*n* = 13). The report was sent to 100 stakeholders of whom 56 viewed the report, but only 13 provided feedback and/or completed the questionnaire related to their stakeholder type at the end. Results included the scores and rankings of policy recommendations and communication guidelines, which were presented using bubble charts with two dimensions: relevance to your organization and feasibility in practice. The differences in scores among the three stakeholder groups were indicated on top of the charts if such difference was significant. The items and the corresponding graphs were displayed in descending order of the ranking, computed by average scores based on relevance and feasibility.

Under the result of each item, there was an open question: “Your comment or feedback in relation to this policy recommendation (e.g., to what extent do you agree with it, how to implement, any possible drawbacks, any other comment related to this item or this outcome of the voting?)”, where stakeholders could comment and voice their opinions. The online evaluation was completed by 13 stakeholders.

### 2.2. Expert Panel

Recruitment was carried out by the European Food Information Council (EUFIC). EUFIC has a wide network of European and international stakeholders covering public policy makers, academia, industry, health professionals, media, etc. Besides the CLYMBOL stakeholder advisory board, various types of European stakeholders, with a direct or indirect involvement in the development, use and regulation of health claims and/or health symbols at the EU level, were invited to participate in the study (Table 1). The sample size was relatively small in the Delphi round 1 and 3, and the distribution of stakeholder type was not equal, because participation of stakeholders was completely voluntary and the incentive was purely based on their professional or personal interests.

### 2.3. Data Analysis

Data analysis was performed using SPSS Statistics 25.0 (IBM SPSS, Armonk, NY, USA). Both means and medians provide meaningful insights and could be employed for different purposes. Means and standard deviations (S.D.) were reported as descriptive statistics since no bimodal distribution has been observed and the medians were often uniform across items. However, medians were used in further analyses instead of means, as medians could be more robust. Friedman non-parametric tests with the Dunn-Bonferroni post hoc method were used to determine if the ranking of scores varied among items.

The distribution of ratings obtained from the Delphi method round 2 was examined and illustrated with bubble charts, and Spearman correlation between relevance and feasibility was calculated for each item. The items were ranked by average score for relevance and feasibility. Scores among the stakeholder groups were compared with Kruskal-Wallis one-way analysis of variance. 

Various consensus criteria exist for the Delphi method. Overall, a high level of consensus can be indicated by highly correlated responses, as several studies have used Cronbach’s α value > 0.90 as a consensus criterion [33,34,35]. In terms of consensus per item, the interquartile range (IQR) measures dispersion of data between the 25th percentile and the 74th percentile [36]. An IQR ≤ 1 shows that more than 50% of scores fall within 1 point on the scale, which is considered a high level of consensus on a 7-point Likert scale [23]. This consensus criterion is also commonly used in Delphi studies with an indicator of IQR ≤ 3 on a 9-point scale [37]. The consensus criteria were not set as priori in this study, and a moderate level of consensus was considered acceptable, since the evaluation criteria were expected to reflect divergence among stakeholders from different types and the outcomes are not critical to immediate health or life as in some previous Delphi studies where a strong consensus was more crucial [38,39]. In this study, items that were selected from the Delphi method round 1 to enter round 2 had an IQR ≤ 4 on the 10-point scales. The items with an IQR ≤ 2 on the 7-point scales in the Delphi method round 2 were considered as having an acceptable level of consensus.

## 3. Results

### 3.1. Compilation of Evidence to Formulate Strategies

Considering the results and evidence drawn from all earlier CLYMBOL studies, an exhaustive list of research findings, policy recommendations, and communication guidelines has been established and presented in Supplementary Material S3. This list constitutes the basis for the Delphi study and the empirical analyses. It is sectioned by the four work areas of the CLYMBOL project. The policy recommendations and communication guidelines are arranged according to the findings where they were derived from.

### 3.2. Evaluation and Priority Setting

#### 3.2.1. Delphi Round 1

**Figure 2:**
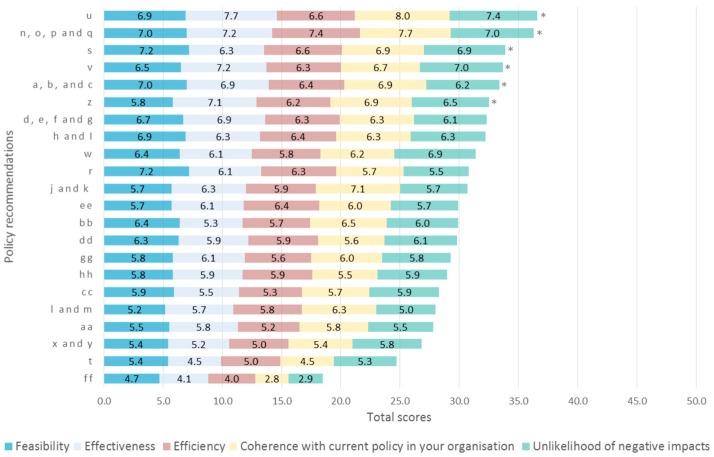
Stakeholder evaluation of policy recommendations based on the five quality criteria (*n* = 10). The vertical axis indicates the numbering of policy recommendations as in Table 2. The horizontal axis denotes the score that each (group of) item(s) received. The total scores range from 5 to 50, but the axis is set to start at 0.0 in order to show the correct proportions of ratings for the quality evaluation criteria. The numbers inside the five sections of each bar correspond with the average scores on the criteria. The total scores do not differ significantly among items based on Friedman non-parametric test (*p*-value = 0.078). The asterisks (*) indicate the items selected for the Delphi round 2.

As the evaluation of findings was not the focus of this study, the results are presented Supplementary material S4. With regards to policy recommendations, Table 2 lists the evaluated (groups of) items and shows the aggregated mean scores based on the five criteria of evaluation. Overall, the policy recommendation **item u** “Focus on ways to improve motivation such as creating information needs and increasing the interest in healthy eating” received the highest ranking across stakeholders. It should be noted that the corresponding finding received the lowest rating (**item 14**, c.f. Supplementary Materials S3 and S4). Stakeholders clearly disapproved of the idea of increasing the prices for products with health symbols (**item ff**). Cronbach’s α based on all items was 0.95, which indicates a high level of consensus. Figure 2 illustrates the scores of each (group of) policy recommendation(s).

**Figure 3:**
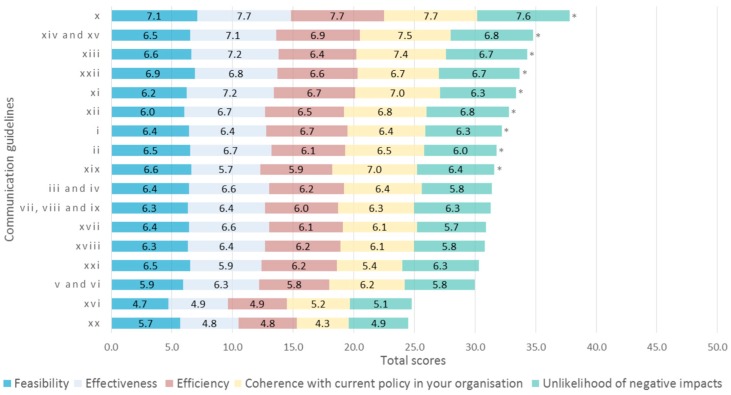
Stakeholder evaluation of communication guidelines based on the five quality criteria (*n* = 10). The vertical axis indicates the numbering of communication guidelines as in Table 2. The horizontal axis denotes the score that each (group of) item(s) received. The total scores range from 5 to 50, but the axis is set to start at 0.0 in order to show the correct proportions of rating for the quality evaluation criteria. The numbers inside the five sections of each bar correspond with the average scores on the criteria. The total scores do not differ significantly among items based on Friedman non-parametric test (*p*-value = 0.080). The asterisks (*) indicate the items selected for the Delphi round 2.

Table 3 lists the evaluated communication guidelines and shows the aggregated mean scores based on the five criteria of evaluation. Overall, the communication guideline **item x** “Keep communication simple and clear, avoid overly complex supporting information that uses scientific and/or regulatory jargon, at the same time limit propositions that are not fully scientifically sound in product positioning and communication strategies” had the highest ranking. This rating is in line with expectations, as the corresponding findings received the highest ratings as well (**items 9** and **10**). The **item xx** “Inform food producers that the consumers from The Netherlands and Denmark are willing to pay the potentially additional costs from improving the healthiness of food products and fulfilling the criteria to bear health symbols” received the lowest ranking, which is in line with the lowest rating of the corresponding policy recommendation (**item ff**). Cronbach’s α based on all items was 0.96, which indicates a high level of consensus. Figure 3 illustrates the scores of each (group of) communication guideline(s).

The items of policy recommendations and communication guidelines with the highest mean scores were selected to enter the Delphi round 2. All (groups of) items had an IQR ≤ 4 except for one communication guideline item (**item x** with IQR = 4.25), but this item was included because it received the highest rating.

#### 3.2.2. Delphi Round 2

The results of Delphi round 2 were presented in Supplementary material S5 using two-dimensional graphs. Supplementary Material S6 shows a summary of the evaluation task based on average scores for relevance, feasibility, and their correlation coefficient (r_s_), which specifies the relationship between the scores on relevance and feasibility. These two criteria are significantly and positively correlated for all items, except for communication guideline **items x** and **xii**. 

For most of the items, scores for relevance and feasibility were similar among the different stakeholder groups. Table 4 summarizes the ranking and statistical tests of all items. Policy recommendation **item u** “Focus on ways to improve motivation such as creating information needs and increasing the interest in healthy eating” and communication guideline **item x** “Keep communication simple and clear, avoid overly complex supporting information that uses scientific and/or regulatory jargon, while at the same time limiting propositions that are not fully scientifically sound in product positioning and communication strategies” were consistently at the top of the ranking. There were some changes in rankings from the Delphi round 1 to round 2; however, the changes could not be meaningfully interpreted, because some items were evaluated in groups in the Delphi round 1 (in order to shorten the very long list), but the grouped items were then divided and evaluated as individual items in the Delphi round 2 (as only a number of items have been selected, there was room for evaluating items individually to provide more accurate insight). As a result, some items shared the same ranking as a group in the Delphi round 1, but the individual items had different rankings in the Delphi round 2. Regarding communication guidelines, **item xiii** “Use innovative ways to communicate the importance of healthy eating, aiming to change the perception of negative association between healthiness and tastiness” shifted from the third place in round 1 to the first place in rounds 2 and 3. The biggest changes in ranking were observed for **items xxii** and **ii**; they shifted from the fourth to the eighth rank and from the eighth to the fourth, respectively. 

Overall, neither the mean scores nor their distribution varied to a large extent (i.e., superscripts in Table 3). In most cases, scores for relevance and feasibility were similar among the stakeholder groups. Although opinions of stakeholders from the same type might also vary, stakeholders from academia had a tendency to give higher scores on feasibility in practice compared to the other stakeholder groups. With regards to consensus measurement, Cronbach’s α based on all policy recommendations was 0.73, and Cronbach’s α based on all communication guidelines was 0.69. These values indicate a moderate level of consensus. Most items had an IQR ≤ 2, except for a few lower-ranked items.

#### 3.2.3. Delphi Round 3

Most stakeholders who participated in the Delphi round 3 did not have further comments about the voting outcomes (*n* = 13). There were a few comments related to the evaluation of policy recommendation **items b** and **q**, where mixed responses were observed. The distribution of scores for relevance and feasibility was bipolar for these two items, in which the amount of stakeholders who rated the items high on the two criteria was comparable to the amount of stakeholders who rated them as low (Supplementary Material S5: Figures S5.10 and S5.11). For **item b**, the comment was “very mixed response to this one. It would be interesting to see which types of organization voted in each way”, and for **item q**, the comment was “again this would be useful to split depending on type of organization—I think that food industry would have a very different view compared to NGO’s on this one”. However, there was no significant difference observed among the three stakeholder groups (i.e., industry, academia and others) regarding the rating of these two items (Table 4). For communication guidelines, there was a comment related to the evaluation of **item xxii** (Supplementary Material S5: Figure S5.19): “health claim legislation was not intended to promote public health, so it is difficult to apply it (**item xxii**) to achieve those aims without strictly having health claims being present or absent on all foods.” A large group of stakeholders rated this item neutral in terms of relevance and feasibility. In general, the Delphi round 3 did not yield additional insight nor changes in the priority list, and thus confirmed the voting outcomes obtained in the Delphi round 2.

## 4. Policy Implications and Conclusion

This study has reviewed a wide range of assessments regarding the role of FOP nutrition labels such as health claims and symbols in food consumer behavior within the CLYMBOL project. It provides a comprehensive list of evidence-based policy recommendations and communication guidelines, and bridges the knowledge and views of stakeholders from various disciplines to evaluate the outcomes. These policy recommendations and communications guidelines are directed towards multiple stakeholder groups and aim to encourage collaboration. By means of an evaluation based on various quality criteria in three Delphi method rounds, qualitative insights were prioritized using quantitative data and policy priorities have been identified. Policy efforts should focus on ways to improve motivation and interest in healthy eating among consumers. This is backed by the findings of [12], wherein motivation was the most important determinant of health claim use among European consumers. Consumers’ interest in healthy eating could be increased by adopting appropriate communication strategies, such as using innovative ways to communicate the importance of healthy eating.

Information campaigns emerge as the most promising while combining a multitude of outreach channels [40]. Public information campaigns are a commonly and frequently used type of intervention to stimulate healthy eating, with some evidence of positive impact in terms of raising awareness, knowledge, and claimed behavior, but without strong evidence of effectiveness in terms of changing nutritional intake and health markers [41]. To date, there has been only limited attention to the way in which various communication channels can complement each other, and concerted use of different information channels could improve the effectiveness of such campaigning. For example, it has been shown that targeted multi-media campaigns aimed at promoting the use of health symbols can affect the prevalence of the health motive while shopping [42]. Meanwhile, there is a clear consensus and strong agreement that communication should be kept simple and clear, yet scientifically sound, as consumers favor health claims with shorter and less complex messages, as well as health symbols with visible endorsement [9,43].

Apart from typical communication, nudging may be an effective way to make health goals more salient or steer consumers to unobtrusively make healthier food choices during their purchase [44,45]. In parallel, product reformulations can also help to change the possible negative association between healthiness and tastiness [46]. Nevertheless, some experts in the study of Khan et al. (2017) reported concern that the health claim regulation may constitute constraints for food product innovation in general [47].

With regard to knowledge, although consumers only have fair knowledge about nutrients and health claims regardless of their education levels [48], knowledge only plays a minor role in shaping health claim use [12]. Therefore, instead of focusing on nutrition-related knowledge, it appears more important and was also generally agreed that consumer awareness about existing health claims and symbols should be increased and consumers should be better informed about their meanings in the context of a healthy diet, as well as about the scope of the EC Regulation 1924/2006. This is also supported by the findings of Sandvik et al. (2018) with regard to the keyhole logo [49], so as to prevent consumers from being misled by unregulated information cues while evaluating products’ healthiness.

While the study results of Edenbrandt et al. (2017) and Smed et al. (2017) suggest that consumers are willing to pay higher prices for products with health symbols [18,20], there was a clear consensus that stakeholders did not agree with the idea of increasing prices for products with health symbols. Despite the fact that scanner data—as used in these studies—provide robust findings against measurement errors, as the data were derived from objective measures of past and actual purchase [50], the policy recommendation and communication guideline stemming from the econometric study of Dutch and Danish household panel data received relatively low ratings. Stakeholders might have considered that increasing prices for products with health symbols would decrease consumer demand and eventually discourage the use of health symbols, and the price differential for products with health symbols indicated by the analysis can be viewed as the value premium the market attaches to products with an improved nutritional profile, but not necessarily as an indicator for the possibility of additional price premiums.

Stakeholders from academia had a tendency to give higher scores on feasibility in practice compared to the other stakeholder groups, which could be explained by the fact that academics are not involved in implementing actual public policy actions. Stakeholders from government, NGOs, legal advisory, consumer organizations, or public health gave a higher feasibility score than industry to the item concerning informing consumers about the EC Regulation 1924/2006, whereby health claims are authorized only when they are substantiated by scientific evidence and proven to be understood and meaningful to average consumers (**item xiv**). This discrepancy could be due to fears of industry about stigma brought on food products without health claims or symbols. On the other hand, the food industry may be able to further exploit marketing opportunity if the ESFA’s approval is highlighted in the communication, as health claims approved by an independent and competent third party tend to be perceived as more credible by consumers [51].

Overall, a moderate consensus was achieved in this Delphi study, which might be the result of the fact that all stakeholders were primed with the same objective at the beginning of the study, i.e. to consider health claims and symbols on food as a means to support consumers in making informed and healthy food choices and foster industry competitiveness, taking into account individual and country differences within the EU, as well as to avoid misunderstanding and undesirable behavioral effects. In line with expectations, a lower level of consensus was observed regarding the criterion “relevance to stakeholder’s organization”, since the stakeholders represented different sectors and disciplines. Disagreement and dissension are sometimes desirable in Delphi studies, as policy issues can be explored in a more creative way, provided that adequate clarification of the different or opposing opinions is available [52]. This marks a potential limitation in the present study, as stakeholders reached consensus relatively easy, and may not have been sufficiently incentivized to further elaborate their ratings in the Delphi round 3.

Similar to other methods of knowledge synthesis, this Delphi study has some limitations in terms of reproducibility and transparency [53]. Participation of stakeholders was completely voluntary and the incentive was purely based on their professional or personal interests. This implies possible self-selection bias from the fact that mostly stakeholders with a high involvement, interest and expectations may have participated in the study. In addition, participants were free to withdraw at any moment during the study. Most stakeholders did not complete all 3 Delphi rounds, nor vote for all items in round 2 systematically, which may have led to a non-response bias. The relatively low degree of active participation in round 3 may have stemmed from a good level of consensus reached already in round 2, as 56 out of the 100 invited participants viewed the results, but only 13 actively provided feedback. Furthermore, the anonymous data do not allow for tracking the evolution of responses of individuals throughout the Delphi study rounds. Hence, it is not possible to investigate changes of opinions of individual stakeholders throughout the study. Besides, there was no information about the food sectors that stakeholders were representing due to anonymity. Participants from different food sectors might respond differently due to their different experiences with health claims and product innovation. In an attempt to control for such limitations, appropriate indications of central tendency, dispersion, and changes of ratings have been provided in this paper. Nevertheless, future Delphi studies may be supplemented with other in-depth quantitative measures with a larger sample size, and possibly in each stakeholder group, to allow a more systematic approach, which enables pseudonymous identification to keep track of the evolution of individual participants and compare responses throughout the study rounds.

In conclusion, this study provides useful insights that guide future policy development aligning consumer protection issues, as well as public health and food marketing communication interests. Meanwhile, it demonstrates how the Delphi method can be an appropriate and effective tool for generating quantitative data to prioritize qualitative insights, so as to set policy priorities for FOP nutrition labels such as health claims and symbols in the EU.

## Figures and Tables

**Table 1:** Types of the participating stakeholders.

Stakeholder Types	Frequency
*Stakeholder workshop for preparation (n = 12)*	
Consumer/Patient Organizations	3
Industry Representation	5
National Authorities	4
*Online survey for Delphi method round 1 (n = 10)*	
Association of Food Industry	2
Consumer Organization	1
Food Industry (Producer/Retailer)	4
Government	2
Health Professional	1
*Stakeholder conference for Delphi method round 2 (n = 100)*	
Academia/Research Institute ^a^	25
Association of Food Industry ^b^	10
Consumer Organization ^c^	3
Food Industry (Producer/Retailer) ^b^	28
Government ^c^	8
Health Professional ^c^	5
Legal Advisor ^c^	7
Non-Governmental Organization (NGO) ^c^	11
Others/No response ^c^	3
*Online report for Delphi method round 3 (n = 13)*	
Academia/Research Institute	2
Association of Food Industry	4
Food Industry (Producer/Retailer)	3
Government	2
Health Professional	1
Non-Governmental Organization (NGO)	1

The superscripts ^a–c^ denote the three stakeholder groups categorized for meaningful statistical comparison in the Delphi round 2: ^a^ academia (*n* = 25); ^b^ industry (*n* = 38); ^c^ others (*n* = 37).

**Table 2:** Policy recommendations ranked based on the aggregated mean scores from the five criteria of evaluation in descending order (*n* = 10).

Policy Recommendations	Mean ± S.D.
u. Focus on ways to improve motivation such as creating information needs and increasing the interest in healthy eating	7.32 ± 2.18
n. Increase consumer awareness about existing health claims and health symbols o. Appoint a national authority or identify the institutes responsible for informing or educating consumers p. Provide accurate information about new or less familiar nutrients of food components for consumers q. Include data on consumer understanding as a generic description in obtaining approval from EFSA	7.26 ± 2.35
s. Call for research on the interaction between information on pack and the individual consumer’s background as to study how consumers interpret the information	6.78 ± 1.94
v. Do not focus only on education or other means to increase objective knowledge about health claims, but also assess consumers’ need for information in this context	6.74 ± 2.77
a. Identify and profile consumer segments to support well-targeted policy actions that also take into account vulnerable groups. b. Appoint a responsible national authority for assessing the impact of health claims and health symbols c. Encourage collaboration between stakeholders, empower them to measure and monitor the effects of health claims and health symbols	6.68 ± 1.64
z. Promote the use of a toolbox of tested methods for various purposes and applications by different stakeholder groups, notably for the use by regulators and industries: To check or document whether a certain health claim/health symbol is understood by the ‘average’ consumer (CUT method) To study how to improve understandability of a health claim/health symbol (laddering method) To investigate whether health claims/health symbols lead to healthier choices (choice experiments) To investigate interactions between health claims/health symbols and context factors (eye-tracking) To study possible negative counter effects in consumption (epidemiological studies or experiments) To study how health claims can be formulated and put into an appropriate context such that they trigger choice (survey together with eye-tracking and laddering) To study which health claims support the company’s CSR policy and/or strengthen brands and corporate image (survey together with laddering)	6.50 ± 2.27
d. Inform consumers about health claims and health symbols with the aim of improving overall understanding of a healthy lifestyle e. Call for consumer research on awareness of, understanding of and attitudes towards health claims and symbols and context factors, possible effects on food choice, purchase and consumption, also take contradictory results and confounding factors into consideration f. Measure the effects on public health (health outcomes or changes in the national health status as a result of the use of health claims and health symbols) g. Analyse the economic impacts in the long term (prevalence, effect on sales, cost-benefit aspects)	6.46 ± 2.07
h. Monitor frequently and coherently health claims and health symbols as well as context factors on the market, analyze the effects of health claims and health symbols in the package context (i.e., color and images) in order to identify gaps in the regulation and use of health claims and health symbols i. Take into account the balance between regulating soft claims and the possible hampering of innovation initiatives	6.44 ± 2.12
w. Increase consumers’ subjective knowledge (perceived confidence) in using health claims especially in countries without health claim regulations prior to 2006	6.28 ± 2.65
r. Encourage the use of health symbols with visible endorsement	6.16 ± 2.74
j. Consider the use of nutrient profile models to regulate nutrition, health claims and symbols, but take into account the possible restrictiveness and extra information load that consumers have to receive. The key is to ensure that the health claims and health symbols fulfil quality standards e.g., certified healthy choice k. Call for research (e.g., modelling studies) which combine information about compositional differences with information about the effects of health claims and health symbols on purchasing and consumption to know whether small differences in nutrient composition of foods with and without health claims and health symbols have any impact (positive or negative) on health	6.14 ± 2.99
ee. Expand the availability of health symbols on various categories (e.g., organic foods) and in different types of stores (e.g., discount stores, small shops outside urban areas, etc.)	5.98 ± 1.69
bb. Create a “health-promoting environment” at the point-of-sale (such as using slogans: “Start the day with a healthy breakfast” or showing pictures of healthy food or people) to prime consumers with a health goal	5.98 ± 1.82
dd. Call for consumer research to identify the underlying reasons for the negative effect of children on the probability of choosing products with health symbols	5.96 ± 2.14
gg. Analyse household scanner data that cover a longer period of time and be aware that the data from an early stage of health symbol introduction could be misleading	5.86 ± 2.06
hh. Do not use BMI alone as the segment criteria for targeting to increase the share of purchasing products with health symbols	5.80 ± 2.57
cc. Call for longitudinal research studies to investigate the possible licensing or other effects of health claims and health symbols covering long term in consumers’ diets	5.66 ± 2.15
l. Harmonise nutrient profiles for health symbols but take into account the differences of public health goals in different countries m. Call for more research efforts to examine the validity of the nutritional criteria of different health symbols	5.60 ± 3.00
aa. Support the use of claim-specific images with sufficient monitoring on the possible misleading effects (e.g., include guidelines in a revised regulation to monitor the use of images on the market)	5.56 ± 2.82
x. Use the taxonomy as a checklist for investigating both desired and undesired effects. y. Analyse the effect of health claims and health symbols in the context in which they are likely to appear and for the target group at which the health claim or health symbol is directed	5.36 ± 2.55
t. Take greater account of public health relevance of health claims, especially food manufacturers and health claim regulators. Health claims should reflect the disease burden in a country; health claims related to conditions which are of low occurrence should be avoided, health claims for diseases with a high burden should be encouraged	4.94 ± 2.51
ff. Increase the prices for products with health symbols (in The Netherlands and Denmark) in order to cover the extra costs of using health symbols or producing healthier food products that fulfil the criteria of using health symbols	3.70 ± 2.57

**Table 3:** Communication guidelines ranked based on the aggregated mean scores from the five criteria of evaluation in descending order (*n* = 10).

Communication Guidelines	Mean ± S.D.
x. Keep communication simple and clear, avoid overly complex supporting information that uses scientific and/or regulatory jargon, at the same time limit propositions that are not fully scientifically sound in product positioning and communication strategies	7.56 ± 2.22
xiv. Inform consumers about the EC Regulation 1924/2006, whereby health claims are authorized only when they are substantiated by scientific evidence and proven to be understood and meaningful to average consumers xv. Use information from sources that are independent and relevant; avoid using low trusted information sources	6.96 ± 1.53
xiii. Use innovative ways to communicate the importance of healthy eating, aiming to change the perception of negative association between healthiness and tastiness	6.86 ±1.71
xxii. Communicate the possible benefits of using health symbols correctly with the aim to increase consumers’ preferences for health symbols	6.74 ± 1.71
xi. Consider that consumers do not interpret health claims and health symbols as experts do, communication should be clearly explaining what health claims and health symbols mean and how they are meant to be used	6.68 ± 1.75
xii. Inform consumers that the prevalence of claims is not necessarily reflective of health priorities; encourage larger communication campaigns, e.g., to explain how health claims (or health symbols) can be relevant for a healthy diet, and what is important when looking after personal health versus when dealing with health issue	6.56 ± 2.70
i. Take into account the needs of different consumer segments and the country-wide differences	6.44 ± 2.05
ii. Provide additional information on product categories bearing health claims and health symbols and the meaning of health claims and health symbols in the context of a balanced diet	6.36 ± 2.63
xix. Communicate health goals at the point-of-sale such as supermarkets	6.32 ± 2.13
iii. Be aware that package design elements such as color, image, logos can be potentially more powerful in communication than scientifically-backed health claims and health symbols iv. Inform consumers about the scope of the EC Regulation 1924/2006 i.e., what is regulated and what is not	6.28 ± 2.31
vii. Make EFSA’s approval process more transparent, and open up communication with consumers and stakeholders, including applicants viii. Use consumer-friendly information (images or texts) to increase familiarity with lesser-known carriers and health effects ix. Include some new or unfamiliar information that may increase attention	6.26 ± 2.10
xvii. Communicate this toolbox of tested methods to different stakeholder groups e.g., through scientific journal papers and types of press releases that reach a wide audience	6.18 ± 1.39
xviii. Convey health message as scientifically-backed health claims and health symbols by using claim-specific images or other context factors such as color to increase clarity and attractiveness	6.16 ± 1.80
xxi. Inform consumers promptly about the introduction or use of health symbols to induce a faster reaction	6.06 ± 1.93
v. Increase or improve communication between the organizations responsible for health symbols vi. Make the nutritional criteria of health symbols clearer and more transparent to consumers, so that they know what health symbols stands for	6.00 ± 2.51
xvi. Use the taxonomy as an inventory of the possibilities for communicating healthfulness to consumers	4.96 ± 2.72
xx. Inform food producers that the consumers from The Netherlands and Denmark are willing to pay the potentially additional costs from improving the healthiness of food products and fulfilling the criteria to bear health symbols	4.90 ± 2.42

**Table 4:** Summary of the evaluation of policy recommendations and communication guidelines based on the Delphi method round 1 and round 2 and confirmed in round 3.

Round 2 (*n* = 100)						Round 1 (*n* = 10)	
Ranking		Mean Scores ^#^	S.D.	IQR	Stakeholder Groups ^‡^	Ranking	Changes
Policy recommendations *							
#1	u. Focus on ways to improve motivation	5.19 ^b,c^	1.15	1.00	-	#1	=
#2	p. Provide accurate information about less familiar nutrients	5.07 ^a,b,c^	1.51	2.00	-	#2	=
#3	z. Promote the use of tested method toolbox	5.07 ^b,c^	1.58	2.00	Relevance: Academia > Others	#6	↑
#4	n. Increase awareness about existing health claims and symbols	5.02 ^c^	1.36	1.00	Relevance: Industry and Academia > Others Feasibility: Academia > Others	#2	↓
#5	a. Profile consumer segments to support well-targeted actions	4.93 ^a,b,c^	1.40	2.00	Feasibility: Academia > Industry	#5	=
#6	s. Call for research on how individual interprets information	4.85 ^a,b,c^	1.45	2.00	-	#3	↓
#7	o. Appoint a national authority for informing consumers	4.83 ^a,b,c^	1.45	2.50	-	#2	↓
#8	v. Focus not only on education but also need for information	4.71 ^a,b,c^	1.42	1.50	Feasibility: Academia > Industry	#4	↓
#9	c. Encourage collaboration between stakeholders and empowerment for monitoring	4.64 ^a,b,c^	1.40	2.00	Feasibility: Industry and Academia > Others	#5	↓
#10	b. Appoint a national authority for impact assessment	4.17 ^a,b^	1.85	3.00	-	#5	↓
#11	q. Include consumer understanding data in EFSA approval process	4.15 ^a^	1.73	3.00	-	#2	↓
Communication guidelines *							
#1/#2	xiii. Use innovative ways to communicate healthy eating	5.20 ^e,f^	1.21	1.50	-	#3	↑
#1/#2	x. Keep communication simple and clear and avoid jargons	5.19 ^f^	1.22	1.50	-	#1	=
#3	xi. Consider that consumers interpret health claims and symbols differently as experts do	4.88 ^e,f^	1.36	1.50	-	#5	↑
#4	ii. Provide additional information in the context of a balanced diet	4.78 ^e,f^	1.37	2.00	-	#8	↑
#5	xiv. Inform consumers about the EC Regulation 1924/2006, avoid using low trusted information sources	4.70 ^d,e,f^	1.57	2.00	Feasibility: Others > Industry	#2	↓
#6	xix. Communicate health goals at the point-of-sale	4.69 ^e,f^	1.64	1.50	-	#9	↑
#7	i. Take into account the needs of different consumer segments	4.59 ^d,e^	1.65	2.50	-	#7	=
#8	xxii. Communicate possible benefits of correct health symbol use	4.27 ^d,e^	1.48	1.50	-	#4	↓
#9	xii. Inform consumers that the prevalence of health claims does not necessarily reflect health priorities	4.04 ^d^	1.33	1.50	-	#6	↓

^#^ Aggregated mean scores based on scores for relevance and feasibility; all minimum scores are 1.00 and maximum scores are 7.00. * Items are listed in short form in this table. The full-length policy recommendations or communication guidelines can be found in Table 2 and Table 3. ^‡^ If scores of relevance and/or feasibility are different among the stakeholder groups at the 0.05 level, a symbol “>“ (greater than) is used to indicate the ranking of groups’ scores for the respective items. The superscripts ^a–c^ (for policy recommendations) or ^e–f^ (for communication guidelines) indicate significantly different ranks of scores at the 0.05 level. This distribution was tested using Friedman non-parametric tests with Dunn-Bonferroni post hoc method, thus it deviated from the overall ranking computed using mean values. For policy recommendations, **item u**, **p**, **z** and **n** shared the same median (5.5), hence the ranking is based on the means. For communication guidelines, the median of **item x** (5.5) is higher than **item xiii** (5) though the difference is not significant, these two items thus share the first and second places at ranking. Symbols “↑” (increased), “↓” (decreased) and “=” (unchanged) denote the changes in ranking of items from the Delphi round 1 to round 2 (and confirmed in round 3).

## Supplementary Materials

The following are available online at http://www.mdpi.com/2072-6643/11/2/403/s1, Supplementary material S1: Template for Implications, recommendations and communication guidelines; Supplementary material S2: CLYMBOL Conference: “Consumers and health claims”; Supplementary material S3: The exhaustive list of findings, policy recommendations and communication guidelines based on the four work areas of the CLYMBOL project; Supplementary material S4: Evaluation of the CLYMBOL study findings from the Delphi method round 1; Supplementary material S5: Evaluation results from the Delphi method round 2; Supplementary material S6: Summary of the evaluation results of policy recommendations and communication guidelines based on mean scores for relevance and feasibility and correlation coefficients.
