# Supplementary material for: Setting Policy Priorities for Front-of-Pack Health Claims and Symbols in the European Union: Expert Consensus Built by Using a Delphi Method
Source: Nutrients. 2019 Feb 14;11(2):403. doi: 10.3390/nu11020403 (PMC6412322; doi:10.3390/nu11020403)
Supplement: Supplementary file 1 [file nutrients-11-00403-s001.zip › Proof_Supplementary Materials_Nutrients-425301/Supplementary Material S1.docx]

**Supplementary material S1.**

**Template for Implications, recommendations and communication guidelines**

**Task:** Identification of implications on the provision of health claims and health-related symbols for different stakeholders

**Work package:** Public Policy Implications: Stimulating Healthy Food Choices for the Consumer and Innovation within the Industry

**Start date:** Month 24; **Duration**: 23 months

**Objectives:** To derive implications of the project results for the different stakeholders in the area of health claims and symbols

**Description of task:** For all relevant stakeholders (consumers, industry, retailers, NGOs, policy makers) implications on the provision of health claims, health-related symbols and their context will be formulated based on the results obtained in WPs 1-4. These implications will be structured according to the taxonomy developed in WPs 1-2. For all elements of the taxonomy and relevant interactions, implications will be derived with regard to supporting informed and healthy choices, and fostering industry competitiveness, taking into account individual and country differences within the EU.

| **TEMPLATE (2-3 PAGES; TO BE COMPLETED BY THE TASK LEADERS)** |
| --- |
| TASK IDENTIFICATION |
| WP (n°, name):  Task (n°, name):  Deliverable (n°, title):  Task leader:  Participant numbers & acronyms: |
| OBJECTIVES |
|  |
| SUMMARY OF FINDINGS |
|  |
| IMPLICATIONS  (structured according to WPs 1-2 taxonomy of health claims, health symbols and their context: see appendix; Note: implications may be extended to nutrition claims if relevant) |
| 1. Identification of general implications |
| 1. Regarding health claims (if possible divided by content, effect direction, effect certainty, framing, target group, (scientific agreement, type of data: cf. Taxonomy) |
|  |
| 1. Regarding health symbols (if possible divided by content, aggregation/ directiveness, target, gradation, scope, information character, endorsement: cf. Taxonomy) |
|  |
| 1. Regarding context factors (mandatory food information, marketing-related information, value-chain information, package design: cf. Taxonomy) |
|  |
| 1. Identification of implications for different stakeholder groups (food industry, retailers, consumers, NGO’s, regulators, public health authorities, health professionals and media/journalists (as far as relevant)) |
| 1. Regarding health claims |
|  |
| 1. Regarding health symbols |
|  |
| 1. Regarding context factors |
|  |
| RECOMMENDATIONS |
| 1. Recommendations on consumer understanding of health claims and symbols |
|  |
| 1. Recommended practices in the provision of health claims and symbols (in their context) that can correctly guide consumers and/or result in desirable effects on purchasing and consumption |
|  |
| 1. Recommendations for the avoidance of misleading consumers and possible undesirable effects of health claims and symbols (in their context) |
|  |
| 1. Recommendations on how to assess the impact of future legislative and voluntary food labelling schemes on understanding, purchasing and consumption for different user groups (users in industry, policy making, and consumer science) |
|  |
| COMMUNICATION GUIDELINES  Note: if applicable: specify guidelines according to information channel (traditional media vs. social media) |
| 1. Guidelines for the effective communication of health claims and symbols (in their context) |
| 1. General |
|  |
| 1. Targeted at consumers (general public) |
|  |
| 1. Targeted at industry/manufacturers/retailers |
|  |
| 1. Targeted at policy makers |
|  |
| 1. Targeted at consumer/patient organisations |
|  |
| 1. Guidelines for supporting information on health claims and symbols |
| 1. General |
|  |
| 1. Targeted at consumers (general public) |
|  |
| 1. Targeted at industry/manufacturers/retailers |
|  |
| 1. Targeted at policy makers |
|  |
| 1. Targeted at consumer/patient organisations |
|  |

Appendix

Fig. 1 - Overview of point of purchase food labelling components (Source: CLYMBOL project)

Fig. 2 – Health claims detailed taxonomy (Source: CLYMBOL project)

Fig. 3 – Symbolic claims detailed taxonomy (Source: CLYMBOL project)

Fig. 4 – Mandatory food information detailed taxonomy (Source: CLYMBOL project)

Fig. 5 – Other information detailed taxonomy (Source: CLYMBOL project)
