# Supplementary material for: Setting Policy Priorities for Front-of-Pack Health Claims and Symbols in the European Union: Expert Consensus Built by Using a Delphi Method
Source: Nutrients. 2019 Feb 14;11(2):403. doi: 10.3390/nu11020403 (PMC6412322; doi:10.3390/nu11020403)
Supplement: Supplementary file 1 [file nutrients-11-00403-s001.zip › Proof_Supplementary Materials_Nutrients-425301/Supplementary material S2.pdf]

claims • symbols • consumers

Role of health-related symbols and claims in consumer behaviour

CLYMBOL stakeholder workshop

CLYMBOL Conference: “Consumers and health claims”

Prof Wim Verbeke, Christine Yung Hung

June 15, 2016 ● Brussels, Belgium

www.clymbol.eu

Overview

- Overall objectives
- Stakeholder survey
- Live voting and results

Overview

- **Overall objectives**
- Stakeholder survey
- Live voting and results

Overall objectives

- To prioritize **policy implications**, **recommendations** and **communication guidelines** identified based on the **findings** from CLYMBOL
- To support consumers in making **informed** and **healthy food choices** and foster **industry competitiveness**, taking into account individual and country differences within the EU
- To **avoid misunderstanding** and **undesirable behavioural effects** on consumers

Overview

- Overall objectives
- **Stakeholder survey**
- Live voting and results

Stakeholder survey

- A list of CLYMBOL **findings**, **implications**, **recommendations** and **communication guidelines** was compiled
- Based on the overall objectives, the exhaustive list of findings, implications, recommendations and communication guidelines were evaluated by stakeholders (n = 10)

Stakeholder group	Frequency
Government	2
Food Industry (Producer / Retailer)	4
Association of Food Industry	2
Consumer Organization	1
Health Professional	1

Stakeholder survey

The 22 (groups of) items of findings were evaluated with a score from 1 – 10 based on five criteria

- **Relevance** (1 = Absolutely irrelevant; 10 = Absolutely relevant)
- **Importance** (1 = Absolutely unimportant; 10 = Absolutely important)
- **Novelty** (1 = Absolutely old finding(s); 10 = Absolutely novel finding(s))
- **Consistency with your own belief** (1 = Absolutely contradictory to belief; 10 = Absolutely consistent with belief)
- **Clarity** (1 = Absolutely unclear; 10 = Absolutely clear)

Stakeholder survey

The 22 (groups of) items of implications and recommendations and 17 (groups of) items of communication guidelines were evaluated with a score from 1 – 10 based on five criteria

- **Feasibility** (1 = Absolutely unfeasible; 10 = Absolutely feasible)
- **Effectiveness** (1 = Absolutely ineffective; 10 = Absolutely effective)
- **Efficiency** (1 = Absolutely inefficient; 10 = Absolutely efficient)
- **Coherence with current policy in your organisation** (1 = Absolutely contradictory to current policies; 10 = Absolutely coherent with current policies)
- **Unlikelihood of negative impacts** (1 = Absolutely likely to have negative impacts; 10 = Absolutely unlikely to have negative impacts)

Stakeholder survey

- Based on the scores given, a **ranking** was assigned to each (group of) item, computed by averaging scores from the five criteria of evaluation
- The **8** (groups of) implications and recommendations and **9** (groups of) communication guidelines (IRC) with the **highest mean scores** will be presented and evaluated in this workshop
- **Mean score** of the IRC item(s) = Score (**Feasibility** + **Effectiveness** + **Efficiency** + **Coherence with current policy in your organisation** + **Unlikelihood of negative impacts**) / **5**

Overview

- Overall objectives
- Stakeholder survey
- **Live voting and results**

Live voting and results

Q1a. Which sector do you represent? Please select the most applicable option.

1. Government
2. Non-Governmental Organization (NGO)
3. Legal Advisor
4. Food Industry (Producer / Retailer)
5. Association of Food Industry
6. Consumer Organization
7. Media
8. Academia / Research Institute
9. Health Professional

Live voting and results

Q1b. How many years of experience do you have in the domain of food or nutrition (policy, industry, science, etc.)?

1. < 5 years
2. 5 - 10 years
3. 11 - 15 years
4. 16 - 20 years
5. > 20 years

Live voting and results

Q1c. How many years of experience do you have in your current stakeholder function?

1. < 5 years
2. 5 - 10 years
3. 11 - 15 years
4. 16 - 20 years
5. > 20 years

Live voting and results

Q2. Did you participate in the online CLYMBOL stakeholder survey?

1. Yes and I have completed the survey
2. Yes but I did not complete the survey
3. No but I have gone through the list attached at the invitation
4. No because I registered within the last two weeks
5. No

Live voting and results

Part 1. Policy implications and recommendations

- Results from stakeholder survey
- Voting on relevance to your organization
- Voting on feasibility in practice

#1 Implication(s) and recommendation(s)

"Focus on ways to improve motivation such as creating information needs and increasing the interest in healthy eating."

Mean score = **7.32**/10

S.D. = 2.18

CLYMBOL

#1 Implication(s) and recommendation(s)

"Focus on ways to improve motivation such as creating information needs and increasing the interest in healthy eating."

Q3a. How would you rate the above implication(s) and recommendation(s) with the score from 1 to 7 in terms of relevance to your organization

Absolutely irrelevant to my organization			Neutral			Absolutely relevant to my organization
◯	◯	◯	◯	◯	◯	◯
1	2	3	4	5	6	7

CLYMBOL

#1 Implication(s) and recommendation(s)

"Focus on ways to improve motivation such as creating information needs and increasing the interest in healthy eating."

Q3b. How would you rate the above implication(s) and recommendation(s) with the score from 1 to 7 in terms of feasibility in practice

Absolutely unfeasible in practice			Neutral			Absolutely feasible in practice
◯	◯	◯	◯	◯	◯	◯
1	2	3	4	5	6	7

CLYMBOL

#2 Implication(s) and recommendation(s)

- "Increase consumer awareness about existing health claims and health symbols."
- "Appoint a national authority or identify the institutes responsible for informing or educating consumer."
- "Provide accurate information about new or less familiar nutrients of food components for consumers."
- "Include data on consumer understanding as a generic description in obtaining approval from EFSA."

Mean score = 7.26/10

S.D. = 2.35

CLYMBOL

#2 Implication(s) and recommendation(s)

"Increase consumer awareness about existing health claims and health symbols."

Q4a. How would you rate the above implication(s) and recommendation(s) with the score from 1 to 7 in terms of relevance to your organization

Absolutely irrelevant to my organization			Neutral			Absolutely relevant to my organization
○	○	○	○	○	○	○
1	2	3	4	5	6	7

CLYMBOL

#2 Implication(s) and recommendation(s)

"Increase consumer awareness about existing health claims and health symbols."

Q4b. How would you rate the above implication(s) and recommendation(s) with the score from 1 to 7 in terms of feasibility in practice?

Absolutely unfeasible in practice			Neutral			Absolutely feasible in practice
○	○	○	○	○	○	○
1	2	3	4	5	6	7

CLYMBOL

#2 Implication(s) and recommendation(s)

"Appoint a national authority or identify the institutes responsible for informing or educating consumers."

Q5a. How would you rate the above implication(s) and recommendation(s) with the score from 1 to 7 in terms of relevance to your organization ?

Absolutely irrelevant to my organization			Neutral			Absolutely relevant to my organization
○	○	○	○	○	○	○
1	2	3	4	5	6	7

CLYMBOL

#2 Implication(s) and recommendation(s)

"Appoint a national authority or identify the institutes responsible for informing or educating consumers."

Q5b. How would you rate the above implication(s) and recommendation(s) with the score from 1 to 7 in terms of feasibility in practice?

Absolutely unfeasible in practice			Neutral			Absolutely feasible in practice
○	○	○	○	○	○	○
1	2	3	4	5	6	7

CLYMBOL

#2 Implication(s) and recommendation(s)

"Provide accurate information about new or less familiar nutrients of food components for consumers."

Q6a. How would you rate the above implication(s) and recommendation(s) with the score from 1 to 7 in terms of relevance to your organization ?

Absolutely irrelevant to my organization			Neutral			Absolutely relevant to my organization
○	○	○	○	○	○	○
1	2	3	4	5	6	7

CLYMBOL

#2 Implication(s) and recommendation(s)

"Provide accurate information about new or less familiar nutrients of food components for consumers."

Q6b. How would you rate the above implication(s) and recommendation(s) with the score from 1 to 7 in terms of feasibility in practice?

Absolutely unfeasible in practice			Neutral			Absolutely feasible in practice
○	○	○	○	○	○	○
1	2	3	4	5	6	7

p. 25 Wim Verbeke
Role of health-related symbols and claims in consumer behaviour www.clymbol.eu

CLYMBOL

#2 Implication(s) and recommendation(s)

"Include data on consumer understanding as a generic description in obtaining approval from EFSA."

Q7a. How would you rate the above implication(s) and recommendation(s) with the score from 1 to 7 in terms of relevance to your organization ?

Absolutely irrelevant to my organization			Neutral			Absolutely relevant to my organization
○	○	○	○	○	○	○
1	2	3	4	5	6	7

p. 26 Wim Verbeke
Role of health-related symbols and claims in consumer behaviour www.clymbol.eu

#2 Implication(s) and recommendation(s)

"Include data on consumer understanding as a generic description in obtaining approval from EFSA."

Q7b. How would you rate the above implication(s) and recommendation(s) with the score from 1 to 7 in terms of feasibility in practice?

Absolutely unfeasible in practice			Neutral			Absolutely feasible in practice
○	○	○	○	○	○	○
1	2	3	4	5	6	7

#3 Implication(s) and recommendation(s)

"Call for research on the interaction between information on pack and the individual consumer's background as to how consumers interpret the information."

Mean score = **6.78**/10

S.D. = 1.94

#3 Implication(s) and recommendation(s)

"Call for research on the interaction between information on pack and the individual consumer's background as to how consumers interpret the information."

Q8a. How would you rate the above implication(s) and recommendation(s) with the score from 1 to 7 in terms of relevance to your organization ?

Absolutely irrelevant to my organization			Neutral			Absolutely relevant to my organization
○	○	○	○	○	○	○
1	2	3	4	5	6	7

#3 Implication(s) and recommendation(s)

"Call for research on the interaction between information on pack and the individual consumer's background as to how consumers interpret the information."

Q8b. How would you rate the above implication(s) and recommendation(s) with the score from 1 to 7 in terms of feasibility in practice?

Absolutely irrelevant to my organization			Neutral			Absolutely relevant to my organization
○	○	○	○	○	○	○
1	2	3	4	5	6	7

CLYMBOL

#4 Implication(s) and recommendation(s)

“Do not focus only on education or other means to increase objective knowledge about health claims, but also assess consumers’ need for information in this context.”

Mean score = **6.74**/10

S.D. = 2.77

CLYMBOL

#4 Implication(s) and recommendation(s)

“Do not focus only on education or other means to increase objective knowledge about health claims, but also assess consumers’ need for information in this context.”

Q9a. How would you rate the above implication(s) and recommendation(s) with the score from 1 to 7 in terms of relevance to your organization ?

Absolutely irrelevant to my organization			Neutral			Absolutely relevant to my organization
○	○	○	○	○	○	○
1	2	3	4	5	6	7

#4 Implication(s) and recommendation(s)

"Do not focus only on education or other means to increase objective knowledge about health claims, but also assess consumers' need for information in this context."

Q9b. How would you rate the above implication(s) and recommendation(s) with the score from 1 to 7 in terms of feasibility in practice?

Absolutely irrelevant to my organization			Neutral			Absolutely relevant to my organization
○	○	○	○	○	○	○
1	2	3	4	5	6	7

#5 Implication(s) and recommendation(s)

- "Identify and profile consumer segments to support well-targeted policy actions that also take into account vulnerable groups."
- "Appoint a responsible national authority for assessing the impact of health claims and health symbols."
- "Encourage collaboration between stakeholders, empower them to measure and monitor the effects of health claims and health symbols."

Mean score = 6.68/10
S.D. = 1.64

#5 Implication(s) and recommendation(s)

"Identify and profile consumer segments to support well-targeted policy actions that also take into account vulnerable groups."

Q10a. How would you rate the above implication(s) and recommendation(s) with the score from 1 to 7 in terms of relevance to your organization ?

Absolutely irrelevant to my organization			Neutral			Absolutely relevant to my organization
○	○	○	○	○	○	○
1	2	3	4	5	6	7

#5 Implication(s) and recommendation(s)

"Identify and profile consumer segments to support well-targeted policy actions that also take into account vulnerable groups."

Q10b. How would you rate the above implication(s) and recommendation(s) with the score from 1 to 7 in terms of feasibility in practice?

Absolutely irrelevant to my organization			Neutral			Absolutely relevant to my organization
○	○	○	○	○	○	○
1	2	3	4	5	6	7

CLYMBOL

#5 Implication(s) and recommendation(s)

"Appoint a responsible national authority for assessing the impact of health claims and health symbols."

Q11a. How would you rate the above implication(s) and recommendation(s) with the score from 1 to 7 in terms of relevance to your organization ?

Absolutely irrelevant to my organization			Neutral			Absolutely relevant to my organization
○	○	○	○	○	○	○
1	2	3	4	5	6	7

CLYMBOL

#5 Implication(s) and recommendation(s)

"Appoint a responsible national authority for assessing the impact of health claims and health symbols."

Q11b. How would you rate the above implication(s) and recommendation(s) with the score from 1 to 7 in terms of feasibility in practice?

Absolutely irrelevant to my organization			Neutral			Absolutely relevant to my organization
○	○	○	○	○	○	○
1	2	3	4	5	6	7

CLYMBOL

#5 Implication(s) and recommendation(s)

"Encourage collaboration between stakeholders, empower them to measure and monitor the effects of health claims and health symbols."

Q12a. How would you rate the above implication(s) and recommendation(s) with the score from 1 to 7 in terms of relevance to your organization ?

Absolutely irrelevant to my organization			Neutral			Absolutely relevant to my organization
○	○	○	○	○	○	○
1	2	3	4	5	6	7

CLYMBOL

#5 Implication(s) and recommendation(s)

"Encourage collaboration between stakeholders, empower them to measure and monitor the effects of health claims and health symbols."

Q12b. How would you rate the above implication(s) and recommendation(s) with the score from 1 to 7 in terms of feasibility in practice?

Absolutely irrelevant to my organization			Neutral			Absolutely relevant to my organization
○	○	○	○	○	○	○
1	2	3	4	5	6	7

CLYMBOL

#6 Implication(s) and recommendation(s)

- Promote the use of a toolbox of tested methods for various purposes and applications by different stakeholder groups, notably for the use by regulators and industries:
  - To check or document whether a certain health claim/health symbol is understood by the 'average' consumer (CUT method)
  - To study how to improve understandability of a health claim/health symbol (laddering method)
  - To investigate whether health claims/health symbols lead to healthier choices (choice experiments)
  - To investigate interactions between health claims/health symbols and context factors (eye-tracking)
  - To study possible negative counter effects in consumption (epidemiological studies or experiments)
  - To study how health claims can be formulated and put into an appropriate context such that they trigger choice (survey together with eye-tracking and laddering)
  - To study which health claims support the company's CSR policy and/or strengthen brands and corporate image (survey together with laddering)

Mean score = 6.50/10 S.D. = 2.27

CLYMBOL

#6 Implication(s) and recommendation(s)

"Promote the use of a toolbox of tested methods for various purposes and applications by different stakeholder groups, notably for the use by regulators and industries:

- (Different applications of CUT method, laddering, choice experiments, eye-tracking, epidemiological studies or experiments, etc.)"

Q13a. How would you rate the above implication(s) and recommendation(s) with the score from 1 to 7 in terms of relevance to your organization

Absolutely irrelevant to my organization			Neutral			Absolutely relevant to my organization
○	○	○	○	○	○	○
1	2	3	4	5	6	7

#6 Implication(s) and recommendation(s)

"Promote the use of a toolbox of tested methods for various purposes and applications by different stakeholder groups, notably for the use by regulators and industries:

- (Different applications of CUT method, laddering, choice experiments, eye-tracking, epidemiological studies or experiments, etc.)"

Q13b. How would you rate the above implication(s) and recommendation(s) with the score from 1 to 7 in terms of feasibility in practice

Absolutely unfeasible in practice			Neutral			Absolutely feasible in practice
○	○	○	○	○	○	○
1	2	3	4	5	6	7

Live voting and results

Part 2. Communication guidelines

- Results from stakeholder survey
- Voting on relevance to your organization
- Voting on feasibility in practice

#1 Communication guidelines

"Keep communication simple and clear, avoid overly complex supporting information that uses scientific and/or regulatory jargon, at the same time limit propositions that are not fully scientifically sound in product positioning and communication strategies."

Mean score = **7.56**/10
S.D. = 2.22

#1 Communication guidelines

"Keep communication simple and clear, avoid overly complex supporting information that uses scientific and/or regulatory jargon, at the same time limit propositions that are not fully scientifically sound in product positioning and communication strategies."

Q14a. How would you rate the above communication guideline(s) with the score from 1 to 7 in terms of relevance to your organization

Absolutely irrelevant to my organization			Neutral			Absolutely relevant to my organization
○	○	○	○	○	○	○
1	2	3	4	5	6	7

#1 Communication guidelines

"Keep communication simple and clear, avoid overly complex supporting information that uses scientific and/or regulatory jargon, at the same time limit propositions that are not fully scientifically sound in product positioning and communication strategies."

Q14b. How would you rate the above communication guideline(s) with the score from 1 to 7 in terms of feasibility in practice

Absolutely unfeasible in practice			Neutral			Absolutely feasible in practice
○	○	○	○	○	○	○
1	2	3	4	5	6	7

#2 Communication guidelines

- "Inform consumers about the EC Regulation 1924/2006, whereby health claims are authorized only when they are substantiated by scientific evidence and proven to be understood and meaningful to average consumers."
- "Use information from sources that are independent and relevant; avoid using low trusted information sources."

Mean score = 6.96/10
S.D. = 1.53

#2 Communication guidelines

- "Inform consumers about the EC Regulation 1924/2006, whereby health claims are authorized only when they are substantiated by scientific evidence and proven to be understood and meaningful to average consumers."
- "Use information from sources that are independent and relevant; avoid using low trusted information sources."

Q15a. How would you rate the above communication guideline(s) with the score from 1 to 7 in terms of relevance to your organization

Absolutely irrelevant to my organization			Neutral			Absolutely relevant to my organization
◯	◯	◯	◯	◯	◯	◯
1	2	3	4	5	6	7

#2 Communication guidelines

- "Inform consumers about the EC Regulation 1924/2006, whereby health claims are authorized only when they are substantiated by scientific evidence and proven to be understood and meaningful to average consumers."
- "Use information from sources that are independent and relevant; avoid using low trusted information sources."

Q15b. How would you rate the above communication guideline(s) with the score from 1 to 7 in terms of feasibility in practice

Absolutely unfeasible in practice			Neutral			Absolutely feasible in practice
◯	◯	◯	◯	◯	◯	◯
1	2	3	4	5	6	7

#3 Communication guidelines

"Use innovative ways to communicate the importance of healthy eating, aiming to change the perception of negative association between healthiness and tastiness."

Mean score = **6.86**/10
S.D. = 1.71

#3 Communication guidelines

"Use innovative ways to communicate the importance of healthy eating, aiming to change the perception of negative association between healthiness and tastiness."

Q16a. How would you rate the above communication guideline(s) with the score from 1 to 7 in terms of relevance to your organization ?

Absolutely irrelevant to my organization			Neutral			Absolutely relevant to my organization
○	○	○	○	○	○	○
1	2	3	4	5	6	7

CLYMBOL

#3 Communication guidelines

"Use innovative ways to communicate the importance of healthy eating, aiming to change the perception of negative association between healthiness and tastiness."

Q16b. How would you rate the above communication guideline(s) with the score from 1 to 7 in terms of feasibility in practice?

Absolutely irrelevant to my organization			Neutral			Absolutely relevant to my organization
○	○	○	○	○	○	○
1	2	3	4	5	6	7

CLYMBOL

#4 Communication guidelines

"Communicate the possible benefits of using health symbols correctly with the aim to increase consumers' preferences for health symbols."

Mean score = **6.74**/10

S.D. = 1.71

CLYMBOL

#4 Communication guidelines

"Communicate the possible benefits of using health symbols correctly with the aim to increase consumers' preferences for health symbols."

Q17a. How would you rate the above communication guideline(s) with the score from 1 to 7 in terms of relevance to your organization ?

Absolutely irrelevant to my organization			Neutral			Absolutely relevant to my organization
○	○	○	○	○	○	○
1	2	3	4	5	6	7

CLYMBOL

#4 Communication guidelines

"Communicate the possible benefits of using health symbols correctly with the aim to increase consumers' preferences for health symbols."

Q17b. How would you rate the above communication guideline(s) with the score from 1 to 7 in terms of feasibility in practice?

Absolutely irrelevant to my organization			Neutral			Absolutely relevant to my organization
○	○	○	○	○	○	○
1	2	3	4	5	6	7

CLYMBOL

#5 Communication guidelines

"Consider that consumers do not interpret health claims and health symbols as experts do, communication should be clearly explaining what health claims and health symbols mean and how they are meant to be used."

Mean score = **6.68**/10
S.D. = 1.75

CLYMBOL

#5 Communication guidelines

"Consider that consumers do not interpret health claims and health symbols as experts do, communication should be clearly explaining what health claims and health symbols mean and how they are meant to be used."

Q18a. How would you rate the above communication guideline(s) with the score from 1 to 7 in terms of relevance to your organization

Absolutely irrelevant to my organization			Neutral			Absolutely relevant to my organization
○	○	○	○	○	○	○
1	2	3	4	5	6	7

#5 Communication guidelines

"Consider that consumers do not interpret health claims and health symbols as experts do, communication should be clearly explaining what health claims and health symbols mean and how they are meant to be used."

Q18b. How would you rate the above communication guideline(s) with the score from 1 to 7 in terms of feasibility in practice

Absolutely unfeasible in practice			Neutral			Absolutely feasible in practice
○	○	○	○	○	○	○
1	2	3	4	5	6	7

#6 Communication guidelines

"Inform consumers that the prevalence of claims is not necessarily reflective of health priorities; encourage larger communication campaigns, e.g. to explain how health claims (or health symbols) can be relevant for a healthy diet, and what is important when looking after personal health versus when dealing with health issue."

Mean score = **6.56**/10

S.D. = 2.70

CLYMBOL

#6 Communication guidelines

"Inform consumers that the prevalence of claims is not necessarily reflective of health priorities; encourage larger communication campaigns, e.g. to explain how health claims (or health symbols) can be relevant for a healthy diet, and what is important when looking after personal health versus when dealing with health issue."

Q19a. How would you rate the above communication guideline(s) with the score from 1 to 7 in terms of relevance to your organization

Absolutely irrelevant to my organization			Neutral			Absolutely relevant to my organization
○	○	○	○	○	○	○
1	2	3	4	5	6	7

CLYMBOL

#6 Communication guidelines

"Inform consumers that the prevalence of claims is not necessarily reflective of health priorities; encourage larger communication campaigns, e.g. to explain how health claims (or health symbols) can be relevant for a healthy diet, and what is important when looking after personal health versus when dealing with health issue."

Q19b. How would you rate the above communication guideline(s) with the score from 1 to 7 in terms of feasibility in practice

Absolutely unfeasible in practice			Neutral			Absolutely feasible in practice
○	○	○	○	○	○	○
1	2	3	4	5	6	7

CLYMBOL

#7 Communication guidelines

"Take into account the needs of different consumer segments and the country-wide differences."

Mean score = **6.44**/10

S.D. = 2.05

CLYMBOL

#7 Communication guidelines

"Take into account the needs of different consumer segments and the country-wide differences."

Q20a. How would you rate the above communication guideline(s) with the score from 1 to 7 in terms of relevance to your organization ?

Absolutely irrelevant to my organization			Neutral			Absolutely relevant to my organization
○	○	○	○	○	○	○
1	2	3	4	5	6	7

#7 Communication guidelines

"Take into account the needs of different consumer segments and the country-wide differences."

Q20b. How would you rate the above communication guideline(s) with the score from 1 to 7 in terms of feasibility in practice?

Absolutely irrelevant to my organization			Neutral			Absolutely relevant to my organization
○	○	○	○	○	○	○
1	2	3	4	5	6	7

#8 Communication guidelines

"Provide additional information on product categories bearing health claims and health symbols and the meaning of health claims and health symbols in the context of a balanced diet."

Mean score = 6.36/10
S.D. = 2.63

#8 Communication guidelines

"Provide additional information on product categories bearing health claims and health symbols and the meaning of health claims and health symbols in the context of a balanced diet."

Q21a. How would you rate the above communication guideline(s) with the score from 1 to 7 in terms of <u>relevance</u> to your organization?

Absolutely irrelevant to my organization			Neutral			Absolutely relevant to my organization
○	○	○	○	○	○	○
1	2	3	4	5	6	7

#8 Communication guidelines

"Provide additional information on product categories bearing health claims and health symbols and the meaning of health claims and health symbols in the context of a balanced diet."

Q21b. How would you rate the above communication guideline(s) with the score from 1 to 7 in terms of <u>feasibility</u> in practice?

Absolutely irrelevant to my organization			Neutral			Absolutely relevant to my organization
○	○	○	○	○	○	○
1	2	3	4	5	6	7

CLYMBOL

#9 Communication guidelines

"Communicate health goals at the point-of-sale (such as supermarkets) to prime consumers."

Mean score = **6.32**/10
S.D. = 2.13

CLYMBOL

#9 Communication guidelines

"Communicate health goals at the point-of-sale (such as supermarkets) to prime consumers."

Q22a. How would you rate the above communication guideline(s) with the score from 1 to 7 in terms of relevance to your organization ?

Absolutely irrelevant to my organization			Neutral			Absolutely relevant to my organization
○	○	○	○	○	○	○
1	2	3	4	5	6	7

CLYMBOL

#9 Communication guidelines

"Communicate health goals at the point-of-sale (such as supermarkets) to prime consumers."

Q22b. How would you rate the above communication guideline(s) with the score from 1 to 7 in terms of <u>feasibility</u> in practice?

Absolutely irrelevant to my organization			Neutral			Absolutely relevant to my organization
○	○	○	○	○	○	○
1	2	3	4	5	6	7

CLYMBOL
claims · symbols · consumers

Thank you!

Wim Verbeke
Christine Yung Hung
and the CLYMBOL team

This project has received funding from the European Union's Seventh Framework Programme for research, technological development and demonstration. (Contract n°311963)
