# Supplementary material for: Setting Policy Priorities for Front-of-Pack Health Claims and Symbols in the European Union: Expert Consensus Built by Using a Delphi Method
Source: Nutrients. 2019 Feb 14;11(2):403. doi: 10.3390/nu11020403 (PMC6412322; doi:10.3390/nu11020403)
Supplement: Supplementary file 1 [file nutrients-11-00403-s001.zip › Proof_Supplementary Materials_Nutrients-425301/Supplementary material S3.docx]

Supplementary material S3.

The exhaustive list of findings, policy recommendations and communication guidelines based on the four work areas of the CLYMBOL project.

| **Work areas** | **Findings** | **Policy recommendations** | **Communication guidelines** |
| --- | --- | --- | --- |
| **Work area 1: Current status of health claims and symbols** | 1. Member states had different histories of use and regulation of health claims and health symbols prior to 2006, but views related to health claims and health symbols varied across different stakeholders even from the same country [1] | 1. Identify and profile consumer segments to support well-targeted policy actions that also take into account vulnerable groups 2. Appoint a responsible national authority for assessing the impact of health claims and health symbols 3. Encourage collaboration between stakeholders, empower them to measure and monitor the effects of health claims and health symbols | 1. Take into account the needs of different consumer segments and the country-wide differences |
|  | 1. The most common health claims were nutrient and other function claims (Article 13.1.a and 13.1.b health claims; the most common health claim-bearing food category was food for specific dietary use (infant and baby foods) [2] | 1. Inform consumers about health claims and health symbols with the aim of improving overall understanding of a healthy lifestyle 2. Call for consumer research on awareness of, understanding of and attitudes towards health claims and symbols and context factors, possible effects on food choice, purchase and consumption, also take contradictory results and confounding factors into consideration 3. Measure the effects on public health (health outcomes or changes in the national health status as a result of the use of health claims and health symbols) 4. Analyse the economic impacts in the long term (prevalence, effect on sales, cost-benefit aspects) | 1. Provide additional information on product categories bearing health claims and health symbols and the meaning of health claims and health symbols in the context of a balanced diet |
|  | 1. The majority of nutrition and health claims were on the front-of-pack (FOP) [2] 2. Images of the food itself or its fresh ingredients were often present and appeared as “soft claims” e.g. freshness and naturalness. The popularity of “soft claims” use may raise concerns about creating misleading health perceptions [2] | 1. Monitor frequently and coherently health claims and health symbols as well as context factors on the market, analyse the effects of health claims and health symbols in the package context (*i.e.* colour and images) in order to identify gaps in the regulation and use of health claims and health symbols 2. Take into account the balance between regulating soft claims and the possible hampering of innovation initiatives | 1. Be aware that package design elements such as colour, image, logos can be potentially more powerful in communication than scientifically-backed health claims and health symbols 2. Inform consumers about the scope of the EC Regulation 1924/2006 *i.e.* what is regulated and what is not |
|  | 1. Foods that carry nutrition claims and/or health claims and/or health symbols have, on average, a marginally better nutritional composition than foods that do not carry such claims or symbols [3] | 1. Consider the use of nutrient profile models to regulate nutrition, health claims and symbols, but take into account the possible restrictiveness and extra information load that consumers have to receive. The key is to ensure that the health claims and health symbols fulfil quality standards *e.g.* certified healthy choice 2. Call for research (*e.g.* modelling studies) which combine information about compositional differences with information about the effects of health claims and health symbols on purchasing and consumption to know whether small differences in nutrient composition of foods with and without health claims and health symbols have any impact (positive or negative) on health | See ii. |
|  | 1. There were low levels of agreement between the nutritional criteria underlying studied health symbols; little variation between countries but large variation within food categories. This questions the ability of these systems to identify ‘healthier’ foods [4] | 1. Harmonise nutrient profiles for health symbols but take into account the differences of public health goals in different countries 2. Call for more research efforts to examine the validity of the nutritional criteria of different health symbols | 1. Increase or improve communication between the organizations responsible for health symbols 2. Make the nutritional criteria of health symbols clearer and more transparent to consumers, so that they know what health symbols stands for |
| **Work area 2: Consumer needs and wants** | 1. Consumer acceptance of health claims was primarily and positively impacted by their familiarity with the nutrient or substance mentioned [5] 2. A familiar claim increased belief in the products’ healthiness and a higher purchase intention (Work area 2) [5], but an extremely familiar claim decreased attention. Yet, consumers’ gaze behaviours (from eye-tracking experiments) were inconsistent with their verbal responses (from questionnaires) (Work area 4) [6] | 1. Increase consumer awareness about existing health claims and health symbols 2. Appoint a national authority or identify the institutes responsible for informing or educating consumers 3. Provide accurate information about new or less familiar nutrients of food components for consumers 4. Include data on consumer understanding as a generic description in obtaining approval from EFSA | 1. Make EFSA’s approval process more transparent, and open up communication with consumers and stakeholders, including applicants 2. Use consumer-friendly information (images or texts) to increase familiarity with lesser-known carriers and health effects 3. Include some new or unfamiliar information that may increase attention |
|  | 1. Health symbols with visible endorsement (*e.g.* an image of a happy tooth within the tooth-friendly symbol) were favoured [5, 7] 2. Consumers favoured shorter and less complex health claims [5, 7] | 1. Encourage the use of health symbols with visible endorsement   See n. and q. | 1. Keep communication simple and clear, avoid overly complex supporting information that uses scientific and/or regulatory jargon, at the same time limit propositions that are not fully scientifically sound in product positioning and communication strategies   See vii. and viii. |
|  | 1. Consumers did not only draw the intended inferences from health claims but also inferences beyond the stated information, based upon personal belief [5] 2. Distinction between different types of claims was not always obvious in consumers’ mind, claim effects were based on the totality of information on pack, framed by consumers’ previous experience and knowledge and hence is idiosyncratic [8] | See h. and n.   1. Call for research on the interaction between information on pack and the individual consumer’s background as to study how consumers interpret the information | See i.   1. Consider that consumers do not interpret health claims and health symbols as experts do, communication should be clearly explaining what health claims and health symbols mean and how they are meant to be used |
|  | 1. Health claims are not strongly related to the health needs of the population which implies that they can be misleading even though they are scientifically accurate [9, 10] | 1. Take greater account of public health relevance of health claims, especially food manufacturers and health claim regulators. Health claims should reflect the disease burden in a country; health claims related to conditions which are of low occurrence should be avoided, health claims for diseases with a high burden should be encouraged | 1. Inform consumers that the prevalence of claims is not necessarily reflective of health priorities; encourage larger communication campaigns, *e.g.* to explain how health claims (or health symbols) can be relevant for a healthy diet, and what is important when looking after personal health versus when dealing with health issue |
|  | 1. Motivation to process health claims rather than the ability to process health claims determines the use of health claims [11] | 1. Focus on ways to improve motivation such as creating information needs and increasing the interest in healthy eating | 1. Use innovative ways to communicate the importance of healthy eating, aiming to change the perception of negative association between healthiness and tastiness   See xix. |
|  | 1. More objective knowledge does not support the use of health claims, which may imply that people who are more knowledgeable, are less motivated to process health claims because they feel less need for (more) information and/or find health claims either irrelevant or even misleading [11] | 1. Do not focus only on education or other means to increase objective knowledge about health claims, but also assess consumers’ need for information in this context | See i. and x. |
|  | 1. Consumers’ ability to process health claims is positively impacted by their level of subjective nutrition and health knowledge [11] 2. Countries without health claims regulations prior to 2006 were characterized by a lower level of subjective and objective nutrition and health knowledge [11] | 1. Increase consumers’ subjective knowledge (perceived confidence) in using health claims especially in countries without health claim regulations prior to 2006 | 1. Inform consumers about the EC Regulation 1924/2006, whereby health claims are authorized only when they are substantiated by scientific evidence and proven to be understood and meaningful to average consumers 2. Use information from sources that are independent and relevant; avoid using low trusted information sources |
|  | 1. Taxonomy of health claim, health symbol and their context cannot be a basis for prediction of effects of health claims and health symbols on consumers [12] | 1. Use the taxonomy as a checklist for investigating both desired and undesired effects 2. Analyse the effect of health claims and health symbols in the context in which they are likely to appear and for the target group at which the health claim or health symbol is directed | 1. Use the taxonomy as an inventory of the possibilities for communicating healthfulness to consumers |
| **Work area 3: Methodological toolbox** | 1. For measuring understanding, the CUT method allows to classify consumers with regard to making safe, risky or vague inferences, to test claims and symbols in context; this method can be used with large samples and is relatively cost-efficient. Laddering allows to trace inferences and follow consumers’ line of thought and therefore give insights into how and why different versions of a claim lead to different inferences [13, 14] 2. For measuring effects on purchasing, actual purchase data are not always available, and controlling for other factors affecting purchase (in addition to the claim) is another barrier. Choice experiments and surveys measuring buying intention can give approximations to actual choice. Eye-tracking is a very useful complementary technique, as it measures attention to claims [6] 3. For measuring effects on consumption, experiments (especially when they rely on single exposure to claims) are suitable only when there is a hypothesis on a specific, strong counter-effect. Otherwise, epidemiological studies are more appropriate, but it can be applied only once the claims has been on the market for some time [15] | 1. Promote the use of a toolbox of tested methods for various purposes and applications by different stakeholder groups, notably for the use by regulators and industries:  - To check or document whether a certain health claim/health symbol is understood by the ‘average’ consumer (CUT method) - To study how to improve understandability of a health claim/health symbol (laddering method) - To investigate whether health claims/health symbols lead to healthier choices (choice experiments) - To investigate interactions between health claims/health symbols and context factors (eye-tracking) - To study possible negative counter effects in consumption (epidemiological studies or experiments) - To study how health claims can be formulated and put into an appropriate context such that they trigger choice (survey together with eye-tracking and laddering) - To study which health claims support the company’s CSR policy and/or strengthen brands and corporate image (survey together with laddering) | 1. Communicate this toolbox of tested methods to different stakeholder groups *e.g.* through scientific journal papers and types of press releases that reach a wide audience |
| **Work area 4: The effects on consumers** | 1. Both implicit packaging cues (colour, pictures) and explicit packaging cues (health claims, health symbols) played an important role in consumer perceptions of healthiness and indulgence of foods [16] 2. Clarity and attractiveness of health communi-cation was higher for claim-specific images in some product categories [17] 3. The same package colour within the product line (across different flavours) was less appreciated by consumers, although it increased product choices based on the health claims (while products with the same heath claims were presented in the same package colour) [17] 4. Foods in light coloured packages are perceived as less calorie-rich, heavy and fattening and more natural, regardless of the presence of satiety claims or satiety symbols [17] | 1. Support the use of claim-specific images with sufficient monitoring on the possible misleading effects (*e.g.* include guidelines in a revised regulation to monitor the use of images on the market)   See e. and h. | 1. Convey health message as scientifically-backed health claims and health symbols by using claim-specific images or other context factors such as colour to increase clarity and attractiveness   See iii. |
|  | 1. The relevance of a claim, either personally or for a stated population group, played a role in food choices [6] 2. Consumers with a relevant health goal more often recognized and chose products with health claim [6] 3. When consumers had a relevant health goal, overall health images and claim-specific images increased the likelihood of choosing the product [6] 4. When consumers were primed with a health goal, they paid more attention to health claims and had more positive attitudes towards scientific wordings in survey data but not in eye-tracking data [6] | See d. and s.   1. Create a “health-promoting environment” at the point-of-sale (such as using slogans: “Start the day with a healthy breakfast” or showing pictures of healthy food or people) to prime consumers with a health goal | See ii.   1. Communicate health goals at the point-of-sale such as supermarkets |
|  | 1. The presence of health symbol at a food buffet had no evidence of licensing effect on unhealthy food choice nor made any changes in food intake, which could be due to lack of credibility of the health symbol, attention and ability to process the health symbol [18] 2. The presence of a satiety claim on a breakfast food product did not influence food intake at the next meal [19] | 1. Call for longitudinal research studies to investigate the possible licensing or other effects of health claims and health symbols covering long term in consumers’ diets   See f. | See i. and ii. |
|  | 1. There was no typical purchaser of food products with health symbols except for households with children, in which they generally have a lower probability of purchasing products with health symbols across products and countries [20] 2. Urban residents had a higher probability of purchasing certain product categories (*i.e.* yoghurt, margarine, cereal and milk) with health symbols [20] | 1. Call for consumer research to identify the underlying reasons for the negative effect of children on the probability of choosing products with health symbols | See i. and xiii. |
|  | 1. Consumer preferences for other product characteristics (*i.e.* organic, which had a negative correlation with Choices logo) or stores (*i.e.* discount stores, which had a much lower share of products with health symbols) might be a barrier for the use of Choices logo in The Netherlands [20] | 1. Expand the availability of health symbols on various categories (*e.g.* organic foods) and in different types of stores (*e.g.* discount stores, small shops outside urban areas, *etc.*) | See xix. |
|  | 1. Consumers valued the healthiness of food that they consumed and placed additional value on health symbols based on Dutch and Danish scanner data [21] | 1. Increase the prices for products with health symbols (in The Netherlands and Denmark) in order to cover the extra costs of using health symbols or producing healthier food products that fulfil the criteria of using health symbols   See g. | See ii.   1. Inform food producers that the consumers from The Netherlands and Denmark are willing to pay the potentially additional costs from improving the healthiness of food products and fulfilling the criteria to bear health symbols |
|  | 1. Consumers’ value on health symbols may only be evident during a certain period after the introduction of health symbols due to market adaptation on stocks and prices [21] | 1. Analyse household scanner data that cover a longer period of time and be aware that the data from an early stage of health symbol introduction could be misleading | 1. Inform consumers promptly about the introduction or use of health symbols to induce a faster reaction |
|  | 1. Stated preference for health symbol (Keyhole logo) was evidently translated into actual purchase [22] 2. Consumers who purchased foods with higher overall healthiness were also more likely to purchase foods with Keyhole logo [22] 3. BMI did not explain the share of products with Keyhole logo purchased (*i.e.* obese and overweight shoppers are as consistent in their stated and revealed preferences for Keyhole as shoppers with normal BMI) [22] | See a., d., n. and y.   1. Do not use BMI alone as the segment criteria for targeting to increase the share of purchasing products with health symbols | See i., ii. and vi.   1. Communicate the possible benefits of using health symbols correctly with the aim to increase consumers’ preferences for health symbols |

**References**

1. Hieke, S., et al., Country differences in the history of use of health claims and symbols. European Journal of Nutrition & Food Safety, 2016. 6(3): p. 148-168.

2. Hieke, S., et al., Prevalence of nutrition and health-related claims on pre-packaged foods: a five-country study in Europe. Nutrients, 2016. 8(3): p. 137.

3. Kaur, A., et al., The nutritional quality of foods carrying health-related claims in Germany, The Netherlands, Spain, Slovenia and the United Kingdom. 2016, Nature Publishing Group.

4. Kaur, N. and D.P. Singh, Deciphering the consumer behaviour facets of functional foods: A literature review. Appetite, 2017. 112: p. 167-187.

5. Hodgkins, C., et al., Understanding how consumers categorise nutritional labels: a consumer derived typology for front-of-pack nutrition labelling. Appetite, 2012. 59(3): p. 806-817.

6. Groeppel-Klein, A., M. Freichel, and S. Kliebenstein, Awareness and Relevance of Health Claims at the Point of Sale. Advances in Consumer Research, 2017. 45: p. 640-640.

7. Miklavec, K., et al., Front of package symbols as a tool to promote healthier food choices in Slovenia: Accompanying explanatory claim can considerably influence the consumer's preferences. Food Research International, 2016. 90: p. 235-243.

8. Klepacz, N.A., et al., When is an image a health claim? A false-recollection method to detect implicit inferences about products’ health benefits. Health Psychology, 2016. 35(8): p. 898.

9. Kaur, A., M. Rayner, and S. Heike, Do health and nutrition claims meet consumers’ health needs? Agro FOOD Industry Hi Tech, 2016.

10. Hung, Y. and W. Verbeke, Consumer Evaluation, Use and Health Relevance of Health Claims in the European Union. Food Quality and Preference, 2019.

11. Hung, Y., et al., Motivation outweighs ability in explaining European consumers’ use of health claims. Food quality and preference, 2017. 58: p. 34-44.

12. Brown, K., et al., CLYMBOL European survey of nutrient and health claims prevalence–classification issues. International Journal of Community Nutrition, 2015(0 (sup): p. 178-178.

13. Grunert, K.G., Recommendations for the Use of Methodologies. , in The Role of Health-Related Claims and Symbols in Consumer Behavior: Outcomes. 2015.

14. Stancu, V., K.G. Grunert, and L. Lähteenmäki, Consumer inferences from different versions of a beta-glucans health claim. Food Quality and Preference, 2017. 60: p. 81-95.

15. Brown, K., et al., Do health claims affect consumption, as measured in the laboratory or in the field? Proceedings of the Nutrition Society, 2016. 75(OCE3).

16. Simon, J., E. Kemeny I Varga A van Herpen, and A. Palasha, The impact of health claims and health symbols on the attention processes of consumers. NeuroPsychoEconomics Conference Proceedings, 2017. In press.

17. Purnhagen, K.P., E. van Herpen, and E. van Kleef, The potential use of visual packaging elements as nudges-an analysis on the example of the EU health claims regime. Nudging–Possibilities, Limitations and Applications in European Law and Economics, Cham/Heidelberg/New York/Dordrecht/London, 2016.

18. Palascha, K., E. van Kleef, and H.C.M. Van Trijp, The Influence of Health Symbols on Consumption Behavior. The Role of Health-Related Claims and Symbols in Consumer Behavior: The Third Newsletter. . 2016.

19. Bilman, E., E. van Kleef, and H. van Trijp, External cues challenging the internal appetite control system—overview and practical implications. Critical reviews in food science and nutrition, 2017. 57(13): p. 2825-2834.

20. Smed, S., et al., Who is the purchaser of nutrition-labelled products? British Food Journal, 2017. 119(9): p. 1934-1952.

21. Edenbrandt, A.K., S. Smed, and L. Jansen, A hedonic analysis of nutrition labels across product types and countries. European Review of Agricultural Economics, 2017. 45(1): p. 101-120.

22. Edenbrandt, A.K. and S. Smed, Exploring the correlation between self-reported preferences and actual purchases of nutrition labeled products. Food Policy, 2018. 77(C): p. 71-80.
