# Supplementary material for: Setting Policy Priorities for Front-of-Pack Health Claims and Symbols in the European Union: Expert Consensus Built by Using a Delphi Method
Source: Nutrients. 2019 Feb 14;11(2):403. doi: 10.3390/nu11020403 (PMC6412322; doi:10.3390/nu11020403)
Supplement: Supplementary file 1 [file nutrients-11-00403-s001.zip › Proof_Supplementary Materials_Nutrients-425301/Supplementary material S4.pdf]

Supplementary material S4.

Evaluation of the CLYMBOL study findings from the Delphi method round 1

Figure S4.1 illustrates the scores for each (group of) finding(s). Overall, the findings (**item 9**) "Health symbols with visible endorsement (*e.g.* an image of a happy tooth within the tooth-friendly symbol) were favoured"; and (**item 10**) "Consumers favoured shorter and less complex health claims" ranked the highest overall, based on the stakeholders' evaluation. The finding (**item 14**) "Motivation to process health claims rather than the ability to process health claims determines the use of health claims" ranked the lowest. Findings related to the effects on consumers appeared to have higher ratings on novelty in general. The stakeholder groups could not be accurately compared due to the small sample size in this Delphi method round. Cronbach's α based on all items was 0.92, which indicates a high level of consensus.

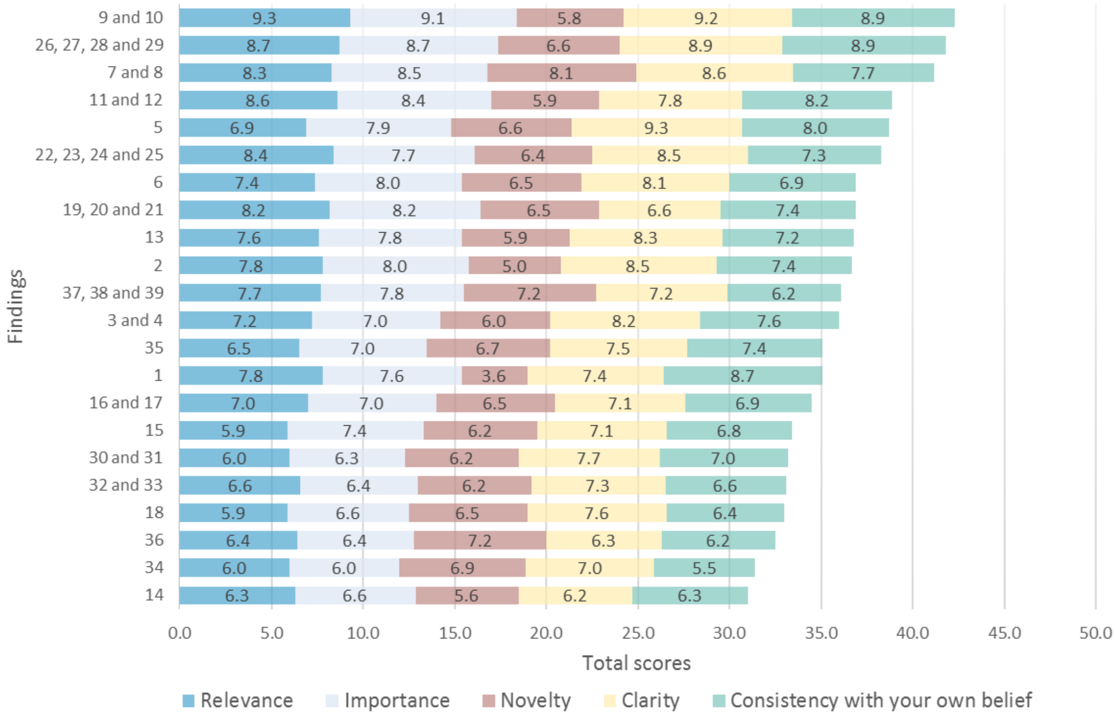

Figure S4. 1 Stakeholder evaluation of findings based on the five quality criteria (n = 10). The vertical axis indicates the numbering of findings from Supplementary material C. The horizontal axis denotes the score that each (group of) item(s) received. The total scores range from 5 to 50, but the axis is set to start at 0.0 in order to show the correct proportions of rating for the quality evaluation criteria. The numbers inside the five sections of each bar correspond with the average scores on the criteria. The total scores do not vary significantly among items based on Friedman non-parametric test (p-value = 0.056).
