# Supplementary material for: Setting Policy Priorities for Front-of-Pack Health Claims and Symbols in the European Union: Expert Consensus Built by Using a Delphi Method
Source: Nutrients. 2019 Feb 14;11(2):403. doi: 10.3390/nu11020403 (PMC6412322; doi:10.3390/nu11020403)
Supplement: Supplementary file 1 [file nutrients-11-00403-s001.zip › Proof_Supplementary Materials_Nutrients-425301/Supplementary material S5.pdf]

Supplementary material S5.

Evaluation results from the Delphi method round 2

S5.1 Policy recommendations

Using policy recommendation **item u** as an example (Figure S5.2), the graph shows the ranking (#) among other policy recommendations, wherein **item u** has been ranked first. The mean score and standard deviation are shown for both dimensions, *i.e.* the horizontal axis represents the scores for relevance to [the participant's own] organisation (5.59 ± 1.49) from 1 to 7 (the higher the score, the more relevant) and the vertical axis represents the scores for feasibility in practice (4.76 ± 1.34) from 1 to 7 (the higher the score, the more feasible). The coordinates of the origin are (4, 4) which is the mid-point of the scale for both relevance and feasibility. The size of the bubble indicates the number of participants who scored an item in a particular way, *i.e.* the more stakeholders gave the same scores, the larger the bubbles are (relative to other bubbles in the same graph). For example, the largest group of stakeholders gave **item u** scores of 6 or 7 for relevance and 5 for feasibility. The correlation coefficient (r_s) specifies the relationship between the scores on relevance and feasibility, wherein the asterisks (* or **) denote the level of statistical significance (*i.e.* p-value < 0.05 or p-value < 0.01, respectively). For **item u**, for example, the relevance is significantly and positively correlated to feasibility.

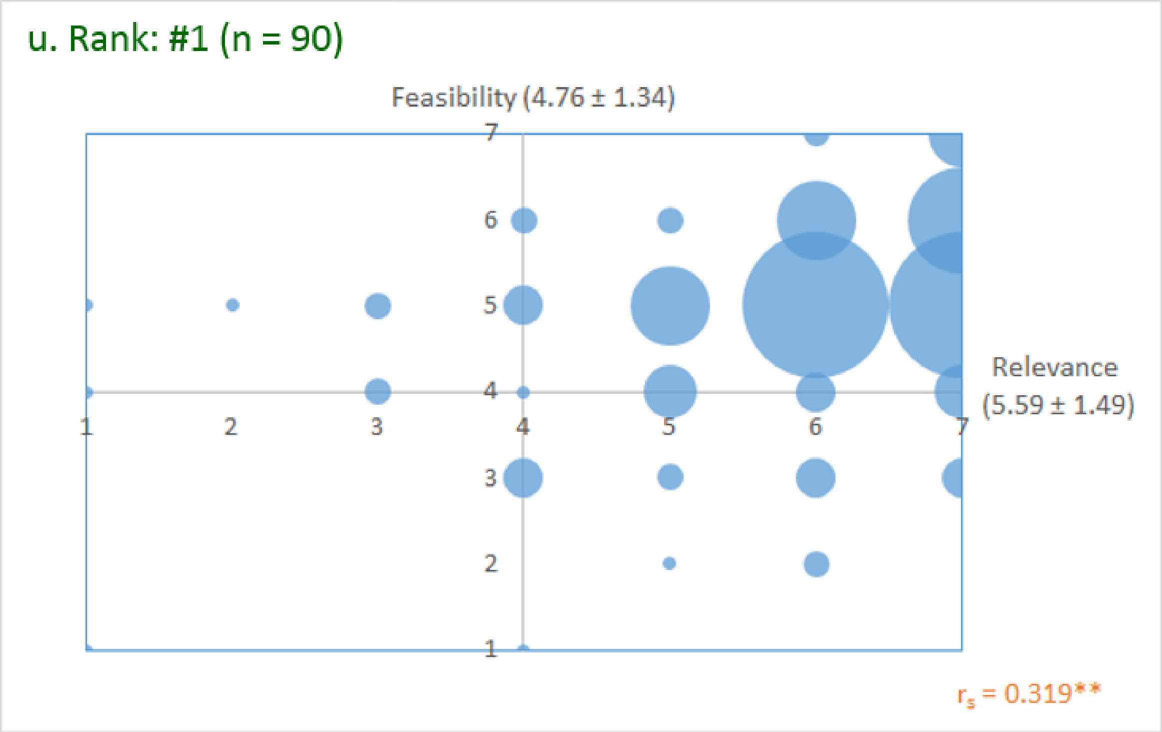

Figure S5. 1 Evaluation of policy recommendation **item u** ("Focus on ways to improve motivation such as creating information needs and increasing the interest in healthy eating") based on two criteria: relevance and feasibility (n = 91)

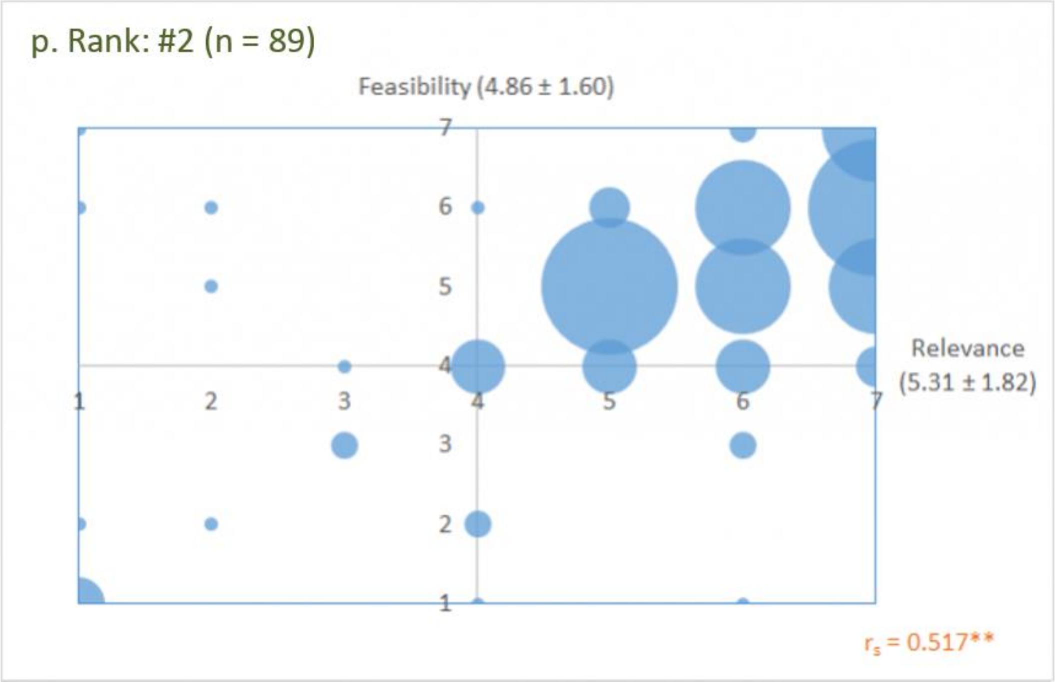

Figure S5. 2 Evaluation of policy recommendation p. based on two criteria (n = 89) (p. Provide accurate information about new or less familiar nutrients of food components for consumers)

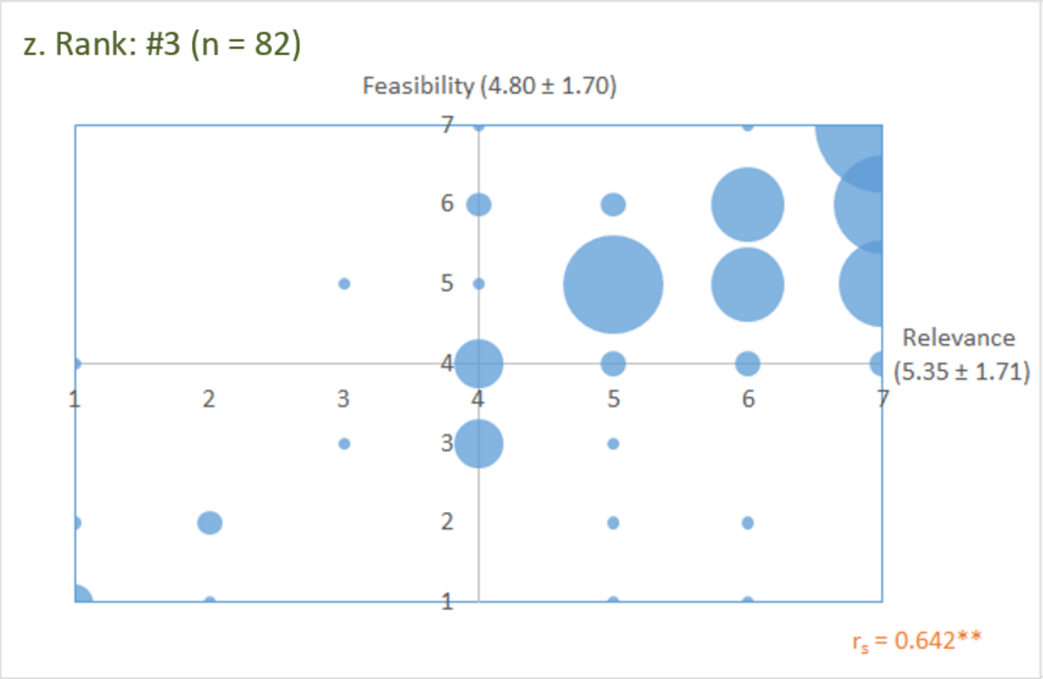

Figure S5. 3 Evaluation of policy recommendation z. based on two criteria (n = 82) (z. Promote the use of a toolbox of tested methods for various purposes and applications by different stakeholder groups, notably for the use by regulators and industries *i.e.* CUT method, laddering, choice experiments, eye-tracking, epidemiological studies or experiments, *etc.*)

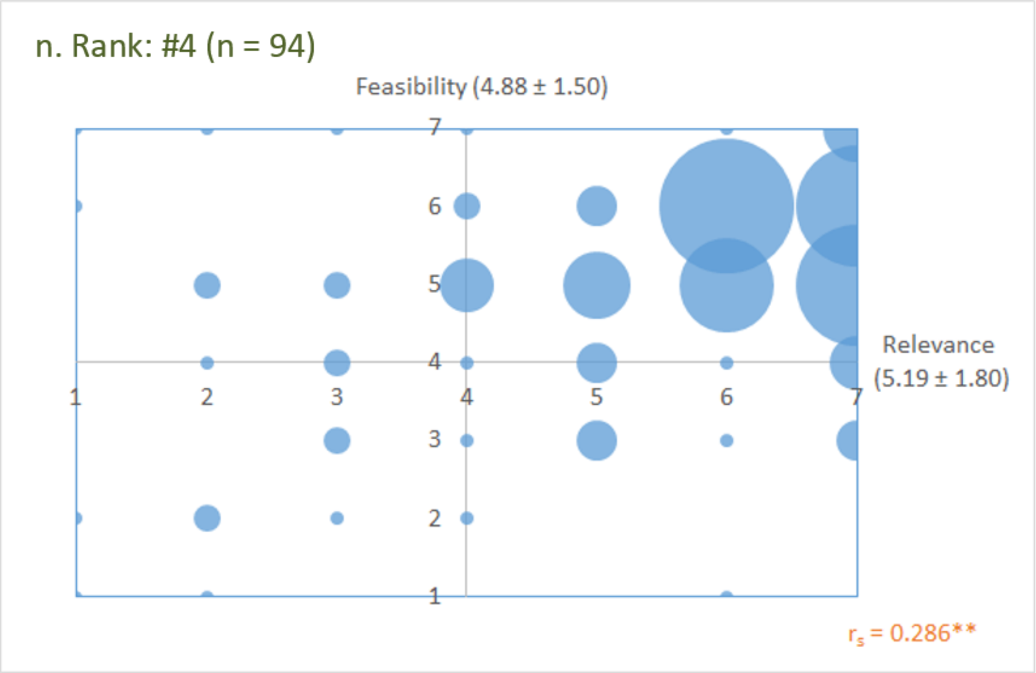

Figure S5. 4 Evaluation of policy recommendation n. based on two criteria (n = 94) (n. Increase consumer awareness about existing health claims and health symbols)

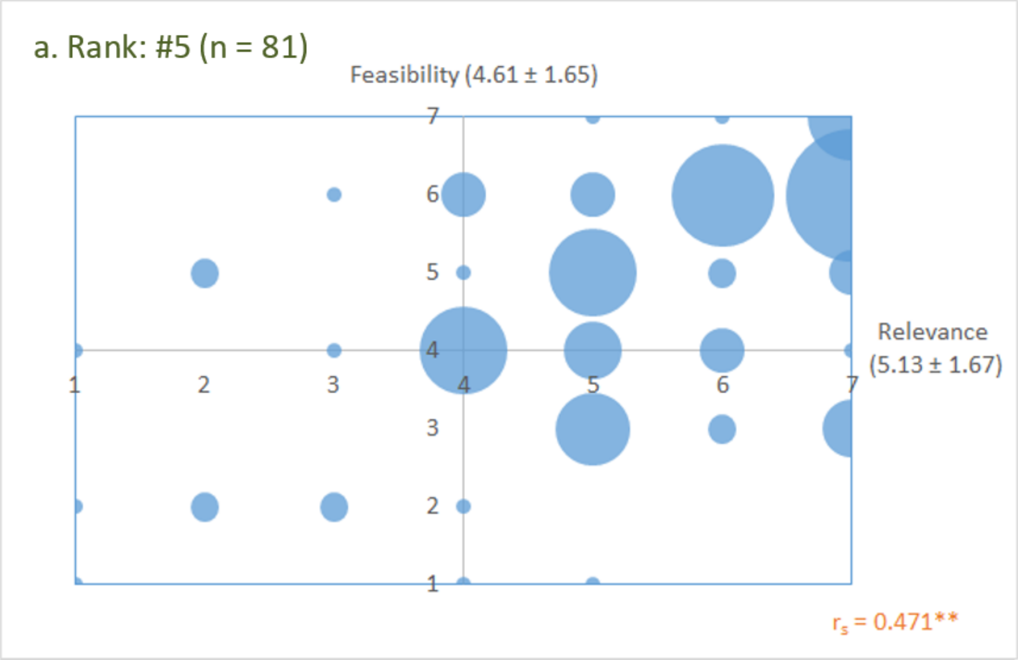

Figure S5. 5 Evaluation of policy recommendation a. based on two criteria (n = 81) (a. Identify and profile consumer segments to support well-targeted policy actions that also take into account vulnerable groups)

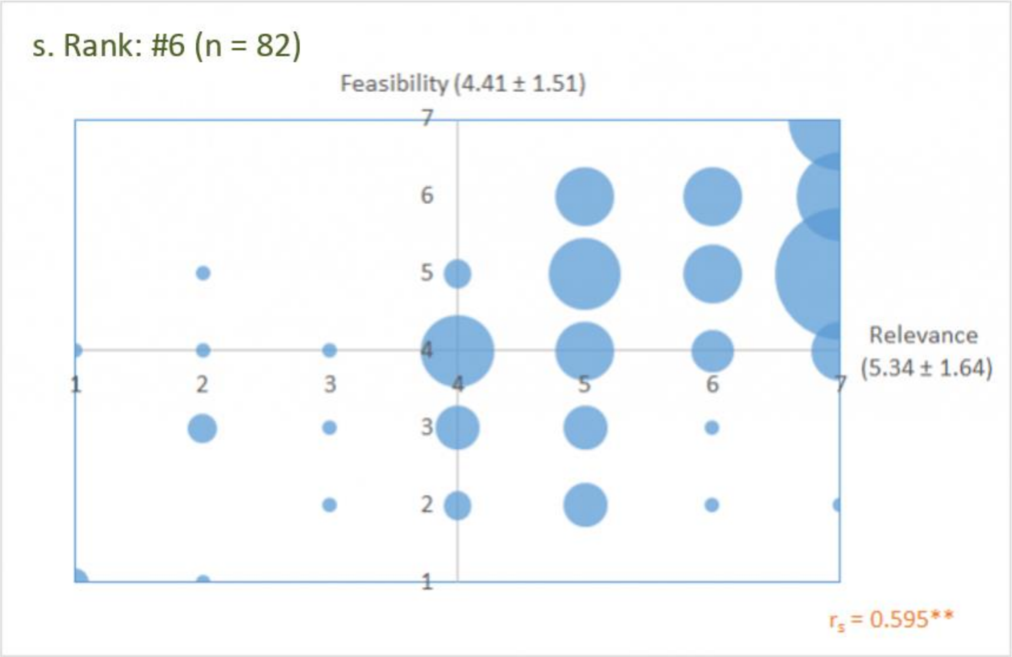

Figure S5. 6 Evaluation of policy recommendation s. based on two criteria (n = 82) (s. Call for research on the interaction between information on pack and the individual consumer's background as to how consumers interpret the information)

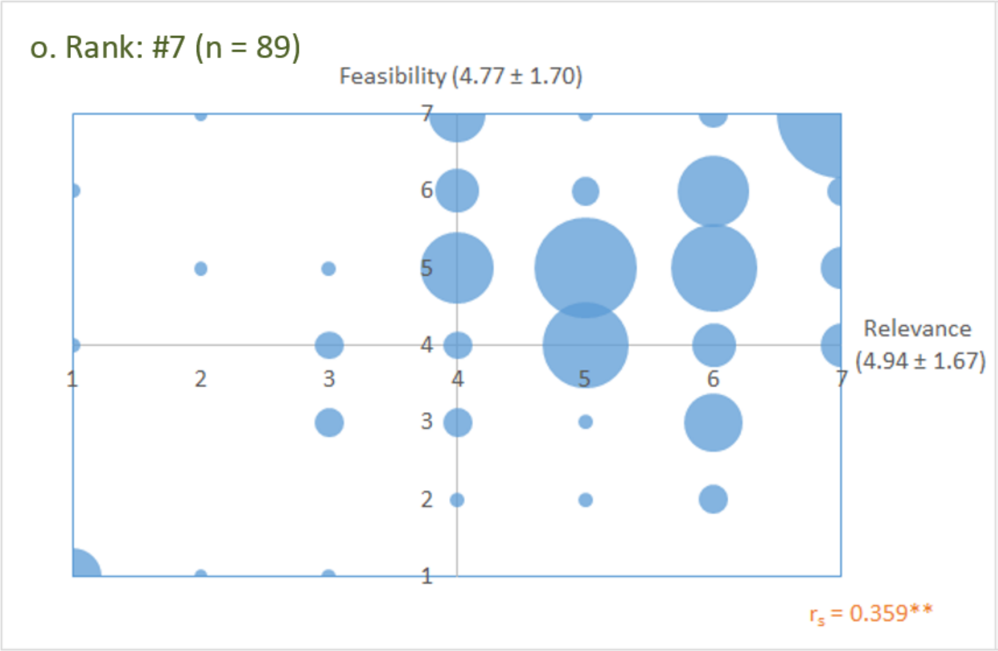

Figure S5. 7 Evaluation of policy recommendation o. based on two criteria (n = 89) (o. Appoint a national authority or identify the institutes responsible for informing or educating consumers)

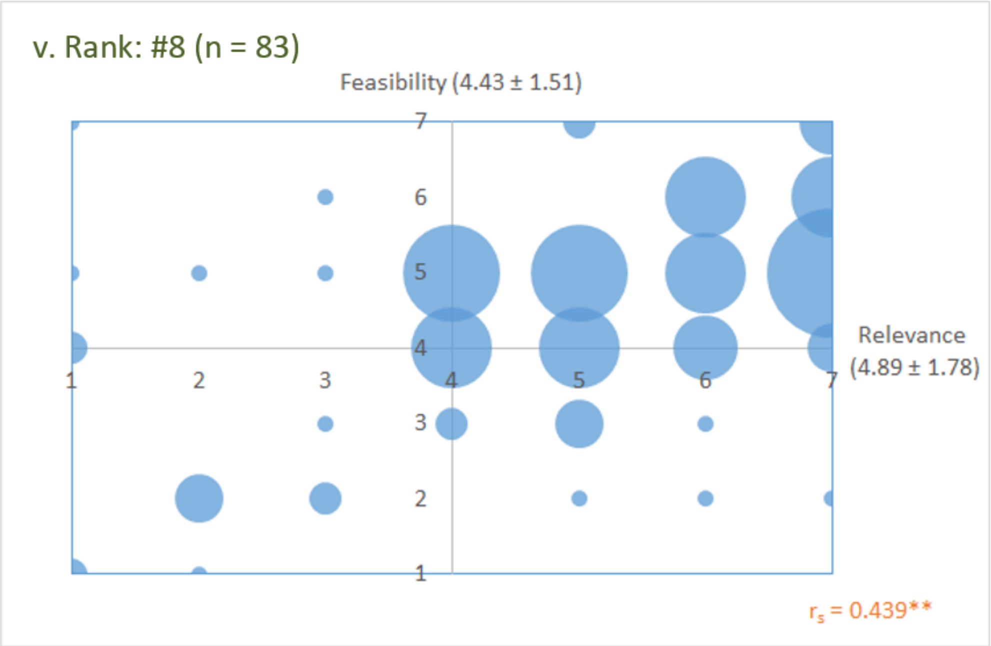

Figure S5. 8 Evaluation of policy recommendation v. based on two criteria (n = 83) (v. Do not focus only on education or other means to increase objective knowledge about health claims, but also assess consumers' need for information in this context)

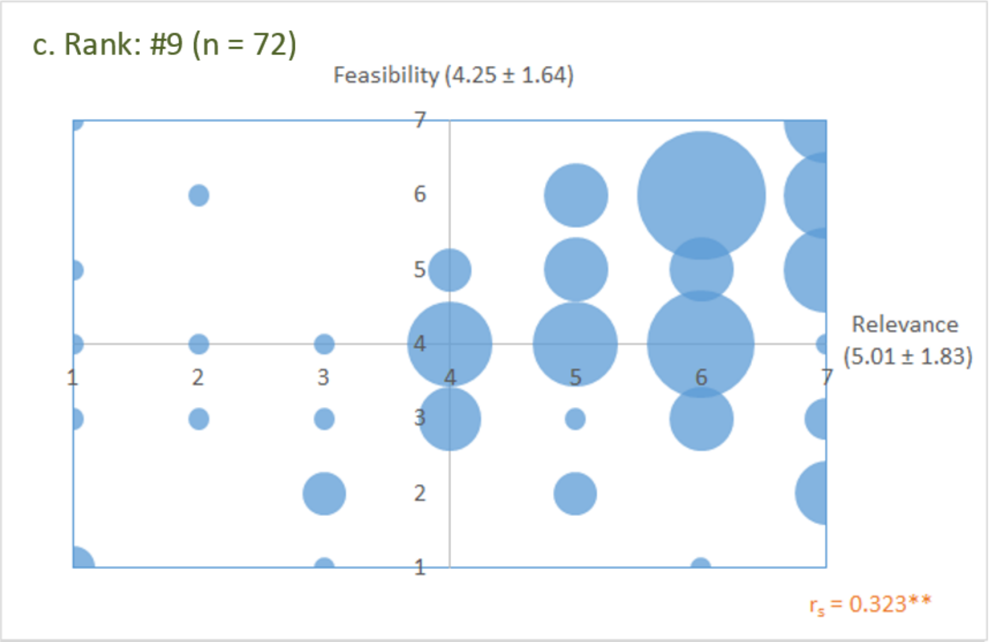

Figure S5. 9 Evaluation of policy recommendation c. based on two criteria (n = 72) (c. Encourage collaboration between stakeholders. Empower them to measure and monitor the effects of health claims and health symbols (IR 10)

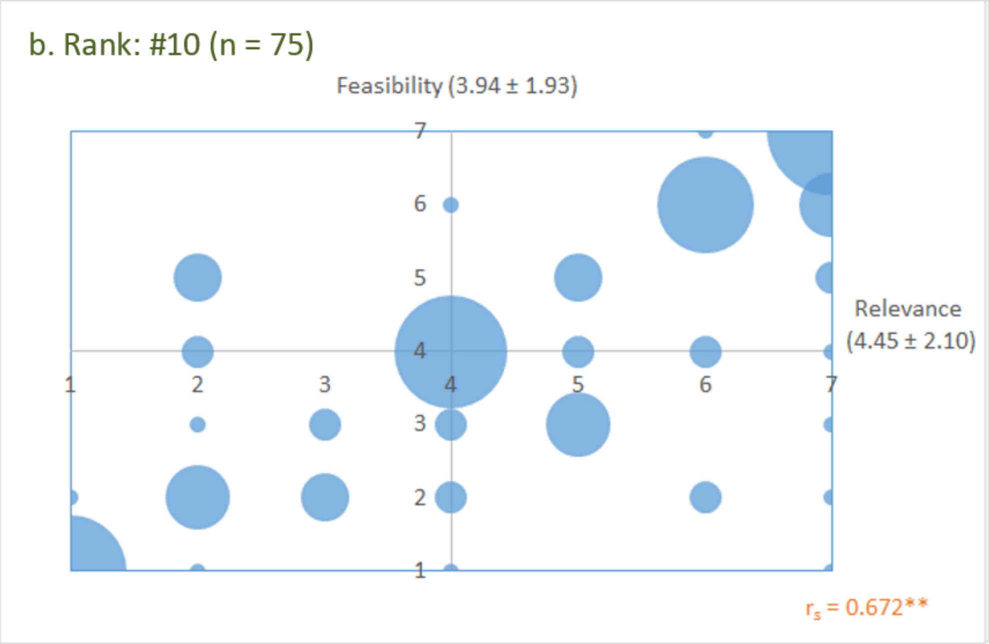

Figure S5. 10 Evaluation of policy recommendation b. based on two criteria (n = 75) (b. Appoint a responsible national authority for assessing the impact of health claims and health symbols)

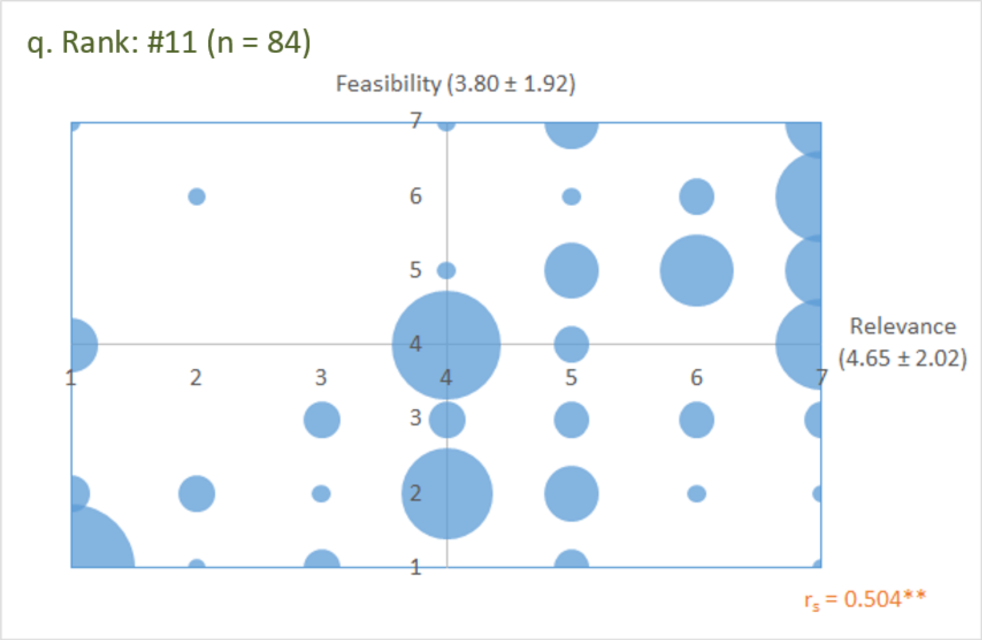

Figure S5. 11 Evaluation of policy recommendation q. based on two criteria (n = 84) (q. Include data on consumer understanding as a generic description in obtaining approval from EFSA)

S5.2 Communication guidelines

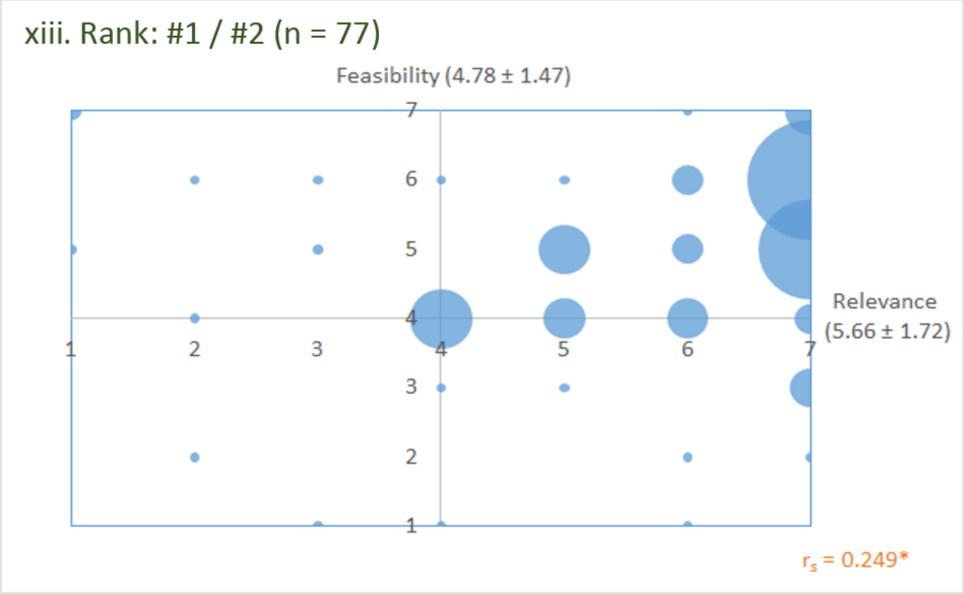

Figure S5. 12 Evaluation of communication guideline xiii. based on two criteria (n = 77) (xiii. Use innovative ways to communicate the importance of healthy eating, aiming to change the perception of negative associations between healthiness and tastiness)

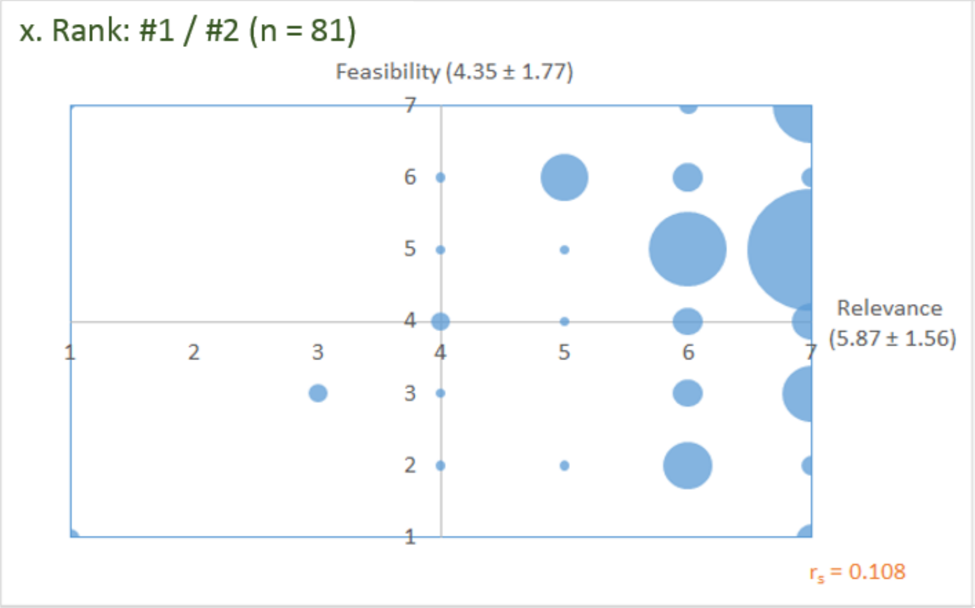

Figure S5. 13 Evaluation of communication guideline x. based on two criteria (n = 81) (x. Keep communication simple and clear, avoid overly complex supporting information that uses scientific and/or regulatory jargon. At the same time limit propositions that are not fully scientifically sound in product positioning and communication strategies)

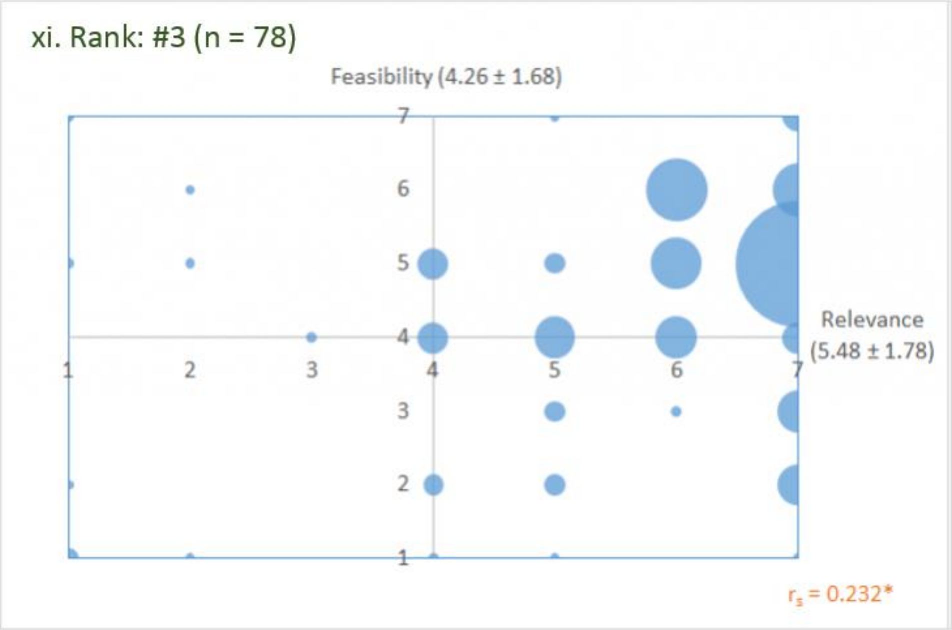

Figure S5. 14 Evaluation of communication guideline xi. based on two criteria (n = 78) (xi. Considering that consumers do not interpret health claims and health symbols as experts do, communication should be clearly explaining what health claims and health symbols mean and how they are meant to be used)

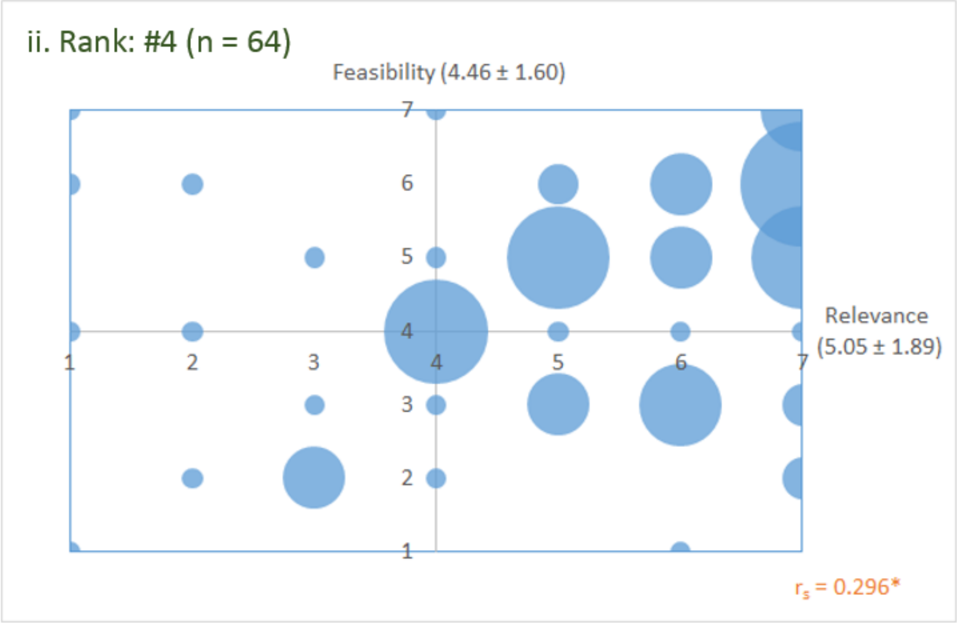

Figure S5. 15 Evaluation of communication guideline ii. based on two criteria (n = 64) (ii. Provide additional information on product categories bearing health claims and health symbols and the meaning of health claims and health symbols in the context of a balanced diet)

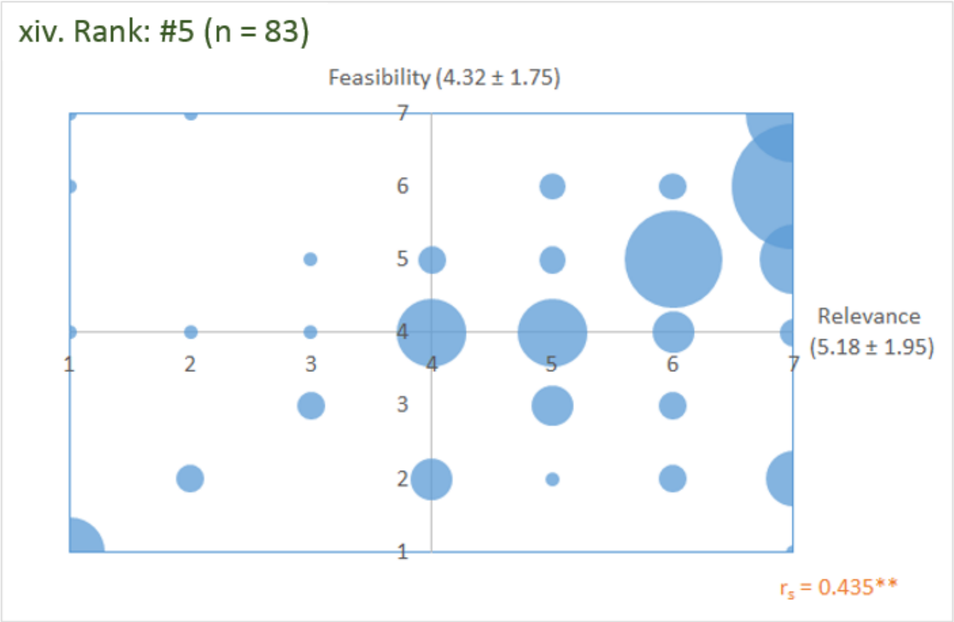

Figure S5. 16 Evaluation of communication guideline xiv. based on two criteria (n = 83) (xiv. Inform consumers about the EC Regulation 1924/2006, whereby health claims are authorised only when they are substantiated by scientific evidence and proven to be understood and meaningful to average consumers. Use information from sources that are independent and relevant. Avoid using low trusted information sources)

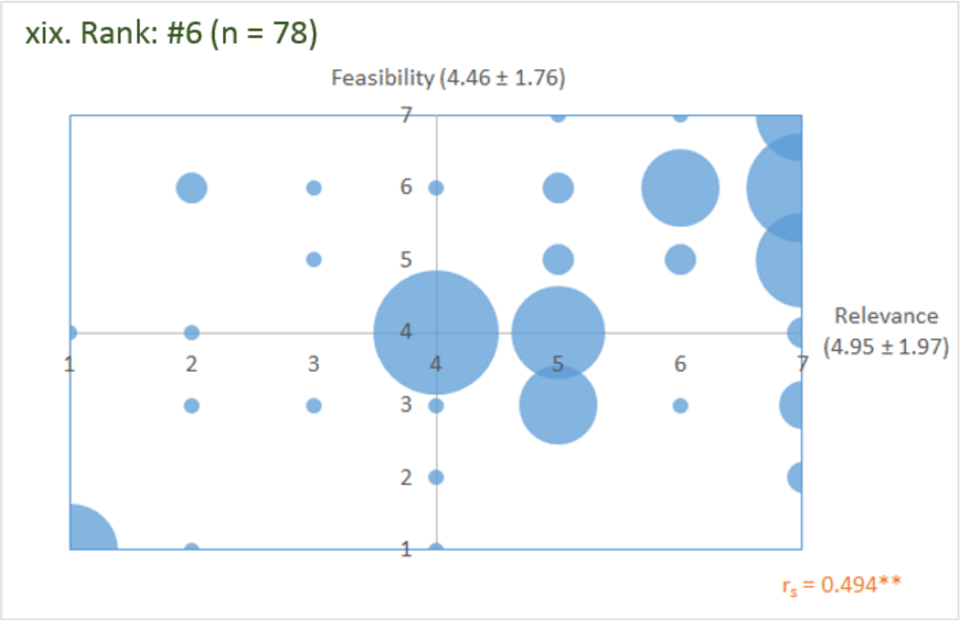

Figure S5. 17 Evaluation of communication guideline xix. based on two criteria (n = 78) (xix. Communicate health goals at the point-of-sale (such as supermarkets) to prime consumers)

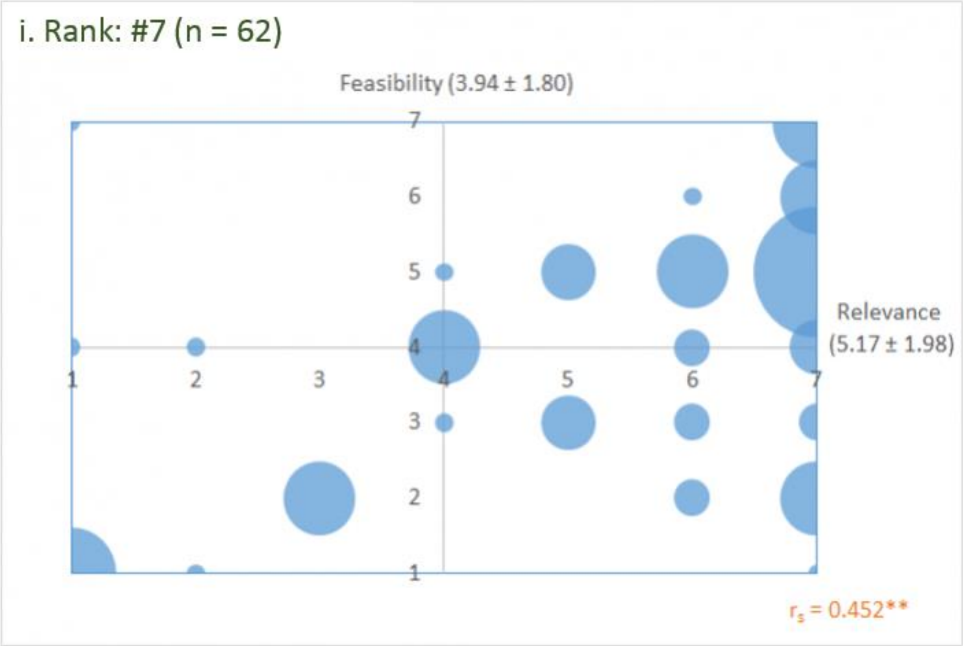

Figure S5. 18 Evaluation of communication guideline i. based on two criteria (n = 62) (i. Take into account the needs of different consumer segments as well as the country-wide differences)

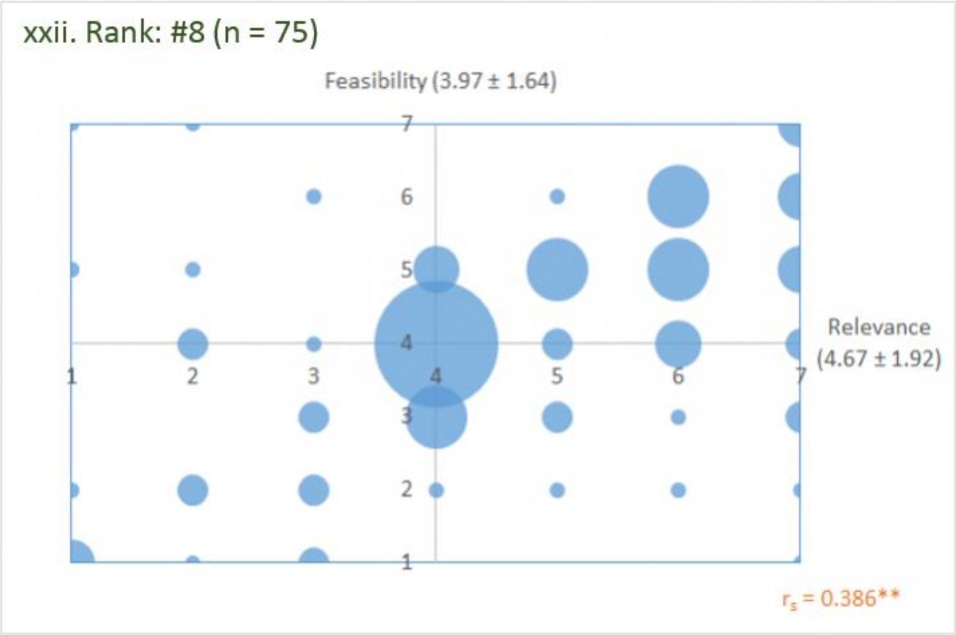

Figure S5. 19 Evaluation of communication guideline xxii. based on two criteria (n = 75) (xxii. Communicate the possible benefits of using health symbols correctly with the aim to increase consumers' preferences for health symbols)

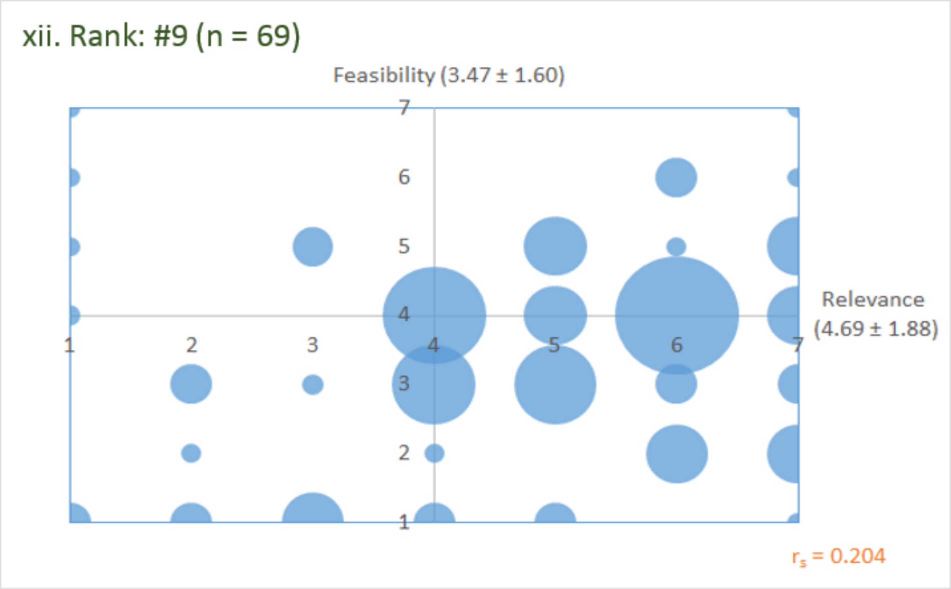

Figure S5. 20 Evaluation of communication guideline xii. based on two criteria (n = 69) (xii. Inform consumers that the prevalence of claims is not necessarily reflective of health priorities. Encourage larger communication campaigns, *e.g.* to explain how health claims (or health symbols) can be relevant)
