# Supplementary material for: Setting Policy Priorities for Front-of-Pack Health Claims and Symbols in the European Union: Expert Consensus Built by Using a Delphi Method
Source: Nutrients. 2019 Feb 14;11(2):403. doi: 10.3390/nu11020403 (PMC6412322; doi:10.3390/nu11020403)
Supplement: Supplementary file 1 [file nutrients-11-00403-s001.zip › Proof_Supplementary Materials_Nutrients-425301/Supplementary material S6.pdf]

Supplementary material S6.

Summary of the evaluation results of policy recommendations and communication guidelines based on mean scores for relevance and feasibility and correlation coefficients

Ranking of items[#]		Mean ± S.D.		Correlation coefficient (r_s)
		Relevance	Feasibility	
**Policy recommendations**				
#1	u. Focus on ways to improve motivation	5.59 ± 1.49	4.76 ± 1.34	0.319**
#2	p. Provide accurate information about less familiar nutrients	5.31 ± 1.83	4.86 ± 1.60	0.517**
#3	z. Promote the use of tested method toolbox	5.35 ± 1.71	4.80 ± 1.70	0.642**
#4	n. Increase awareness about existing health claims and symbols	5.19 ± 1.80	4.88 ± 1.50	0.286**
#5	a. Profile consumer segments to support well-targeted actions	5.13 ± 1.67	4.61 ± 1.65	0.471**
#6	s. Call for research on how individual interprets information	5.34 ± 1.64	4.41 ± 1.51	0.595**
#7	o. Appoint a national authority for informing consumers	4.94 ± 1.67	4.77 ± 1.70	0.359**
#8	v. Focus not only on education but also need for information	4.89 ± 1.78	4.43 ± 1.51	0.439**
#9	c. Encourage collaboration between stakeholders and empowerment for monitoring	5.01 ± 1.83	4.25 ± 1.64	0.323**
#10	b. Appoint a national authority for impact assessment	4.45 ± 2.10	3.94 ± 1.93	0.672**
#11	q. Include consumer understanding data in EFSA approval process	4.46 ± 2.02	3.80 ± 1.92	0.504**
**Communication guidelines**				
#1 / #2	xiii. Use innovative ways to communicate healthy eating	5.66 ± 1.72	4.78 ± 1.47	0.249*
#1 / #2	x. Keep communication simple and clear and avoid jargons	5.87 ± 1.56	4.35 ± 1.77	0.108
#3	xi. Consider that consumers interpret health claims and symbols differently as experts do	5.48 ± 1.78	4.26 ± 1.68	0.232*
#4	ii. Provide additional information in the context of a balanced diet	5.05 ± 1.89	4.46 ± 1.60	0.296*
#5	xiv. Inform consumers about the EC Regulation 1924/2006, and avoid using low trusted information sources	5.18 ± 1.95	4.32 ± 1.75	0.435**
#6	xix. Communicate health goals at the point-of-sale	4.95 ± 1.97	4.46 ± 1.76	0.494**
#7	i. Take into account the needs of different consumer segments	5.17 ± 1.98	3.94 ± 1.80	0.452**
#8	xxii. Communicate possible benefits of correct health symbol use	4.67 ± 1.92	3.97 ± 1.64	0.386**
#9	xii. Inform consumers that the prevalence of health claims does not necessarily reflect health priorities	4.69 ± 1.88	3.47 ± 1.60	0.204

[#] Items are listed in short form in this table. The full-length policy recommendations or communication guidelines can be found in Table 2 and Table 3; ** p-value < 0.01; * p-value < 0.05 denote the level of statistical significance.
